# C-FOS promotes the formation of neutrophil extracellular traps and the recruitment of neutrophils in lung metastasis of triple-negative breast cancer

**DOI:** 10.1186/s13046-025-03370-2

**Published:** 2025-03-28

**Authors:** Shuai Yan, Wenxi Zhao, Juntong Du, Lizhi Teng, Tong Yu, Peng Xu, Jiangnan Liu, Ru Yang, Yuhan Dong, Hongyue Wang, Lingran Lu, Weiyang Tao

**Affiliations:** 1 Department of Breast Surgery, The First Afffliated Hospital of Harbin Medical University, Harbin, Heilongjiang 150001 China; 2 Key Laboratory of Hepatosplenic Surgery, Ministry of Education, Harbin Medical University, Harbin, Heilongjiang 150001 China; 3 Key Laboratory of Acoustic, Optical and Electromagnetic Diagnosis and Treatment of Cardiovascular Diseases, Harbin Medical University, Harbin, Heilongjiang 150001 China; 4 The Cell Transplantation Key Laboratory of National Health Commission, Harbin Medical University, Harbin, Heilongjiang 150001 China

**Keywords:** Triple-negative breast cancer, Neutrophil extracellular traps, c-FOS, Neutrophil recruitment, Lung metastasis

## Abstract

**Background:**

Neutrophil extracellular traps (NETs) are composed of DNA chains from neutrophils and associated proteolytic enzymes, which play an important role in cancer metastasis. However, the molecular mechanism of NET-mediated lung metastasis in triple-negative breast cancer (TNBC) remains unclear.

**Methods:**

The expression levels of NETs in breast cancer specimens and serum were analyzed and compared with normal samples. Single-cell sequencing bioinformatics analysis was conducted to identify differentially expressed genes and functional enrichment related to NET formation in patients with breast cancer. The effects of c-FOS on neutrophil recruitment and NET formation in TNBC were investigated. The upstream and downstream regulatory mechanisms mediated by c-FOS were explored through in vitro and in vivo experiments. Therapeutic approaches targeting c-FOS for treating TNBC were further studied.

**Results:**

Inhibition of c-FOS can suppress tumor growth and lung metastasis in TNBC. Mechanistically, c-FOS promotes transcription by binding to the PAD4 promoter region, facilitating the formation of NETs. Additionally, the activation of the ROS-p38 pathway further enhances c-FOS expression. High expression of c-FOS also promotes the expression of inflammatory factors, facilitating neutrophil recruitment. Both in vitro and in vivo experiments demonstrated that the application of T5224 effectively inhibits the formation of NETs, suppressing lung metastasis and tumor growth.

**Conclusion:**

In summary, this study demonstrates that the ROS-p38-cFOS-PAD4 axis can increase NET formation in TNBC and promote the expression of inflammatory factors, facilitating neutrophil recruitment. Therefore, targeting this pathway may help inform new therapeutic strategies and provide new insights for immunotherapy in TNBC.

**Supplementary Information:**

The online version contains supplementary material available at 10.1186/s13046-025-03370-2.

## Background

Breast cancer is the most common malignancy and the world’s second leading cause of cancer-related death in women, which poses a major threat to women’s health and lives [1]. Triple-negative breast cancer (TNBC) represents approximately 20% of breast cancer cases and is characterized by low survival rates, high metastasis rates, and a lack of effective therapeutic targets. Hence, further investigation of the metastatic mechanism of TNBC, especially complex interactions in the tumor microenvironment, has become the focus of current research.

Neutrophils are the most abundant type of white blood cells in the bloodstream and play an important role in the human immune response. Neutrophils can form neutrophil extracellular traps (NETs) that are composed of DNA chains and associated proteolytic enzymes, which are primarily used to capture and eliminate pathogens. However, metastatic breast cancer cells can induce neutrophils to form NETs, thus supporting tumor metastasis [2].

In the process of establishing a pre-metastatic niche, NET-associated neutrophils promote the recruitment of neutrophils with the potential to form NETs by degrading anti-tumor factors such as TSP-1 in the microenvironment, thereby creating favorable conditions for tumor cell metastasis [3]. Moreover, NET-DNA acts as both a trap for cancer cells and a chemotactic factor for attracting these cells [4]. NETs in the liver or lungs have been shown to form distant metastasis by attracting cancer cells [3, 4]. Intercellular interactions within the tumor microenvironment also play a key role in breast cancer metastasis. For example, cysteine protease C (CTSC) secreted by tumor cells regulates the NET formation and neutrophil recruitment to promote lung metastasis of breast cancer [3]. CTSC facilitates NF-kB activation and IL-1β processing via PR3 to further upregulate CCL3 and IL-6, which are involved in neutrophil recruitment. Additionally, the CTSC-PR3-IL-1β axis induces neutrophils to produce reactive oxygen species, further promoting NET formation. NETs can degrade platelet-derived protein-1 and support the metastatic growth of lung cancer cells. In summary, elucidating the mechanism of NET formation and its role in breast cancer metastasis has become an urgent scientific question that needs to be addressed.

Changes in the expression and activity of transcription factors (TFs) are symbols of cancer and play a crucial role in maintaining stem cell functions [5, 6]. Many small molecule regulators targeting tumor-related TFs have been used in clinical trials for cancer treatment [7]. As an oncogene activated in various tumors, c-Fos exerts TF activity by forming a transcription factor AP-1 (activator protein 1) complex with c-JUN through the encoding of nuclear DNA-binding proteins, which is involved in tumor cell proliferation, migration, and therapeutic resistance processes [8]. The role of c-Fos in maintaining cancer stem cells (CSCs) has also been reported in lung cancer, breast cancer, and gliomas [9–11]. AP-1 consists of homologous or heterologous dimers that contain distinct subunits from ATF, Fos, and Jun families [12]. The activity of AP-1 is stimulated by ligands such as carcinogenic stimuli, chemokines and cytokines, thereby regulating many cellular processes. Moreover, it has been shown that SUMOylation, methylation and other post-translational modifications can affect the composition of AP-1 subunits to change transcriptional capacity [13]. The Fos protein is a basic region leucine zipper (bZIP) protein that interacts with Jun or other bZIP proteins to construct the AP-1 complex. Many cell types or tissues express c-Fos constitutively to mediate proliferation, angiogenesis, metastasis and invasion by regulating downstream genes and forming heterodimers with Jun proteins [14, 15]. Fos is crucial for autophagy induction, and its expression level is lower in melanoma tissues than in corresponding non-tumorous tissues [14]. Additionally, transcriptional activation mediated by c-FOS is involved in the onset of diseases and is regulated by PRMT4/CARM1 [16].

This study indicates that c-FOS can promote the progression of TNBC. ChIP and dual-luciferase gene reporter experiments confirm that c-FOS can bind to the PAD4 promoter region to activate transcription, thereby promoting NET formation. The ROS-p38 pathway upregulates the expression of c-FOS and promotes the formation of NETs, thereby contributing to lung metastasis in TNBC. Targeting c-FOS therapy can effectively inhibit tumor growth and lung metastasis. This study may deepen an understanding of the metastatic mechanism of breast cancer and provide an important theoretical foundation and practical guidance for developing new treatment strategies.

## Methods

### Reagents

The phorbol 12-myristate 13-acetate (PMA) was obtained from Sigma-Aldrich (#52440). The recombinant human c-FOS protein was obtained from Abcam (#ab56280). The PAD4 inhibitor GSK484 was obtained from Sigma-Aldrich. The c-FOS inhibitor T5224, the p38 inhibitor SB203580, the p38 agonist Metformin HCl, and the ROS inhibitor Acetylcysteine were all obtained from Selleck Chemicals (Shanghai, China). The DNase I was obtained from Roche.

### Bioinformatics *analysis of single-cell sequencing*

First, corresponding single-cell sequencing data was searched for and downloaded from the Gene Expression Omnibus (https://www.ncbi.nlm.nih.gov/geo/query/acc.cgi). To detect the expression levels of every cell, the R package DropletUtils was employed, filtering out barcodes that did not have cell expression [17]. Following the number of unique molecular identifiers (UMIs) for every cell, further filtering was performed. The scatter package was then utilized to compute gene expression levels, which filtered out cells that had mitochondrial gene expression ratios above 10% and ribosomal gene expression ratios less than 10% [18]. Finally, a statistical analysis of the genes was conducted. For normalizing the expression matrix for the filtered samples, the NormalizeData function from the Seurat package was adopted [19]. Through the utilization of the harmony package, batch effect correction was next carried out [20]. To choose the top 2000 genes that had the most important intercellular differences, the FindVariableFeatures function from the Seurat package was adopted. Focusing on these genes in subsequent analyses aids in highlighting biological signals within the single-cell dataset. To implement linear scaling of the expression data, the ScaleData function from the Seurat package was next employed. By utilizing the RunPCA function from the Seurat package, linear dimensionality reduction analysis (PCA) was ultimately performed. Based on the PCA results, the harmony package was employed to implement batch effect correction [20]. The principal components (PCs) that had high standard deviations were chosen, and the FindNeighbors and FindClusters functions from the Seurat package were utilized to carry out cell clustering analysis. The RunUMAP function from the Seurat package was then utilized to implement non-linear dimensionality reduction analysis (UMAP). Subsequently, to obtain marker genes, the FindMarkers function in the Seurat package was used to calculate various expression genes across clusters. Cells were labeled based on existing marker genes to create clustered visualization plots [21]. Following both the Gene Ontology database and the KEGG PATHWAY DATABASE biochemical pathways database, the functional enrichment analysis of candidate genes was made [22, 23].

### Cell lines and neutrophil extraction

The human breast cancer cell lines MDA-MB-231, HCC1937, MCF-7 and the mouse breast cancer cell line 4T1 were all purchased from ATCC. By utilizing the short tandem repeat (STR) analysis, the cell lines were identified, with negative mycoplasma contamination testing. Through the use of Ficoll density gradient centrifugation, human neutrophils were isolated from the peripheral blood of healthy donors, and magnetic beads (Miltenyi Biotec, 130-104-913) were also utilized to positively choose CD66b^ + ^cells. Each cell was cultured within a 5% CO2 and 37 °C humidified incubator and grown within RPMI 1640 or DMEM that included 10% fetal bovine serum.

### Patient and tissue samples

The immunofluorescence staining was carried out on the primary tumors (which included 30 cases in total) from breast cancer patients at the First Affiliated Hospital of Harbin Medical University between 2023 and 2024. The serum and plasma samples were obtained from breast cancer patients who were hospitalized at that Hospital and recruited healthy volunteers during the same period. In addition, each sample was collected from patients offering informed consent, and each associated process gained approval from the internal review and ethics committee of that Hospital.

### Conditional media

To collect the conditioned media (CM) from cancer cells, the 10 cm culture dishes were used to grow each cell until they were nearly 80% confluent. They were incubated at 37 °C in a serum-free medium for 24 h after they were washed three times by utilizing a serum-free medium at 37 °C. Upon the collection of the CM, it went through centrifugation for 10 min at 2000 g and filtering by employing a 0.22 μm filter, followed by its storage at -80 °C for subsequent uses.

### Immunohistochemical staining (IHC)

Paraffin-embedded samples were sectioned at a thickness of 4 μm. Antigen retrieval was performed using a target retrieval solution with a pH of 9.0 (DAKO), by immersion in a pressure cooker for 15–20 min to remove the aldehyde crosslinks formed during the initial tissue fixation process. Nonspecific binding was blocked using 5% BSA at room temperature for 25 min, followed by overnight incubation of the tissue with anti-CD66B, anti-Ly6G, anti-Ki67, or anti-CD31 antibodies at 4 °C. Immunodetection was performed using DAB (DAKO) according to the manufacturer’s instructions. TUNEL staining was conducted following the manufacturer’s instructions for the TUNEL kit (Shanghai Ruisai Biotechnology Co., Ltd., Shanghai, China).

### NETs detection with SYTOX green

After being seeded on the 96-well plate, neutrophils (100,000) were incubated in serum-free DMEM or CM, where inhibitors were added according to the indications, for 4 h. Then, the SYTOX GREEN (50 nM) was added to the plate, and detection and imaging were performed using a fluorescent inverted microscope after 5 min.

### Immunofluorescence staining

Following the manufacturer’s manual, the tyramide signal amplification (TSA) technique was taken as the basis to carry out Multiplex immunofluorescence staining by utilizing the four-color multiple fluorescent immunohistochemical staining kit (#abs50012, Absin, China). According to what has been mentioned before, when antigen retrieval and blocking were finished, primary antibodies were then incubated at 37 ℃ for 30 to 60 min, and Opal was then applied to incubate HRP-conjugated secondary antibodies and TSA. Each section was finally counterstained through the use of DAPI and was also mounted in the glycerol and gelatin mounting medium.

### ROS analysis

For the purpose of measuring ROS levels, we cultured neutrophils in the cancer cell CM or the non-conditioned medium at 37℃ for 1 h. After incubating the cells with 5 µ mol/L DHE in the dark for 30 min at 37℃, they were washed by using PBS and photographed with a fluorescence inverted microscope or ROS was detected using a reactive oxygen species fluorescence detection kit (Elabscience, Wuhan, China).

### Luminex assay

Multiplex cytokine analysis was performed using Luminex. The conditioned medium from neutrophils co-cultured with breast cancer cells was centrifuged at 400 g at 4 °C for 5 min. Before being utilized, the supernatant was maintained at -80 °C. The analytes measured were as follows: MIP-1β, IL-6, IFN-γ, IL-1ra, IL-5, GM-CSF, TNF-α, RANTES, IL-2, IL-1β, Eotaxin, Basic FGF, VEGF, PDGF-BB, IP-10, IL-13, IL-4, MCP-1, IL-8, MIP-1α, IL-10, G-CSF, IL-15, IL-7, IL-12p70, IL-17, and IL-9. All assays were carried out in accordance with the manufacturer’s instructions. We produced a standard curve and also utilized the Milliplex Analyst software (version 5.1) to decide the concentrations.

### Western blot

After their extraction from cells through employing the RIPA buffer, proteins were separated via the SDS-PAGE, followed by their transformation to the polyvinylidene fluoride (PVDF) membrane. Primary antibodies used included anti-c-FOS (1:1000, CST, 31254), Phospho-p38 (1:1000, CST, 4511), β-ACTIN (1:20,000, ProteinTech, 66009-1-lg), p38 MAPK (1:1000, CST, 8690), PAD4 (1:1000, ProteinTech, 17373-1-AP), and β-Tubulin (1:5000, Abmart, M20005). We utilized a horseradish peroxidase-conjugated secondary antibody (CST), and adopted enhanced chemiluminescence (ECL, Thermo) to detect antigen-antibody reactions.

### RNA isolation and quantitative real-time PCR (RT-PCR)

Total RNA was extracted from cells using TRIzol^®^ reagent (Invitrogen; Thermo Fisher Scientific, Inc.). Subsequently, 0.5 µg of total RNA was reverse transcribed using the PrimeScript RT Reagent Kit (Takara Bio, Inc.). FastStart Universal SYBR Green Master Mix (Roche Applied Science) and gene-specific primers were employed, with GAPDH serving as the internal control. RT-qPCR was performed using the ABI 7500 Fast Real-Time PCR Detection System (Applied Biosystems; Thermo Fisher Scientific, Inc.). To normalize the results, expression levels relative to GAPDH were assessed using the 2–ΔΔCq method. The primer sequences were as follows: PAD4 forward (F), 5’‑GCA CAA CAT GGA CTT CTA CGT GG‑3’ and reverse (R), 5’‑CAC GCT GTC TTG GAA CAC CAC A‑3’; GAPDH forward (F), 5’‑GTC TCC TCT GAC TTC AAC AGC G‑3’ and reverse (R), 5’‑ACC ACC CTG TTG CTG TAG CCA A‑3’. The thermocycling conditions were as follows: initial denaturation at 95˚C for 10 min, followed by 40 cycles of denaturation at 95˚C for 15 s and annealing/extension at 60˚C for 30 s.

### ChIP analysis

Slight modifications were made to the manufacturer’s protocol by utilizing the ChIP detection kit (cat. no. bes5001, Guangzhou Bersinbio Co., Ltd.). The 1% formaldehyde was used to crosslink the cells, and glycine was added to the final concentration of 0.125 M to terminate the reaction. DNA was immunoprecipitated from sonicated cell lysates through the use of c-FOS antibodies (1:100), in which IgG (BD Biosciences) was taken as the negative control. As previously mentioned, RT-qPCR was used to amplify the immunoprecipitated DNA and therefore detect the binding sites of c-FOS. To analyze the amplified fragments, 3% agarose gel electrophoresis was then utilized. Before immunoprecipitation, we took chromatin at a concentration of 10% as the control input. Below were the primer sequences: PAD4 site 1 F, 5’ ACCAGCATTGACACCCATCT 3’ and R, 5’ CAGAGGCCATGAGTCAGCAC − 3’; DANCR site 2 F, 5’ ATGCGGTCAGCCAGAGAAAT 3’ and R, 5’ AGCCCTACAGTGGGGATCTG 3’; and DANCR site 3 F, 5’ TCAAGGTGTTCAGGGCCTCTA 3’ and R, 5’ GTGTGGGCTCTCAAAATCTCC 3’.

### Dual-luciferase reporter assay

GenScript (Nanjing, China) was utilized to construct the PAD4 promoter plasmid. Through the use of GenScript (Nanjing, China), we established the c-FOS wild-type (c-FOS WT) plasmid and a variety of truncation plasmids. By using GenScript (Nanjing, China), the c-FOS promoter point mutation plasmid was established.

After being cultured in 24-well plates, HEK-293T cells underwent transfection by using expression plasmids with different concentrations and a Renilla luciferase control plasmid by employing Lipofectamine 2000 Transfection Reagent (Thermo). 48 h after transfection, a dual luciferase assay (Promega) was used to measure firefly and renilla luciferase activities following the manufacturer’s protocol, in which quantification was carried out on the relative luciferase activity. We experimented three times independently.

### Transwell assay

5 × 10^5 cancer cells were added into the upper chamber (363096, BD). The 1:1 mixture of RPMI 1640 and cancer cell CM, or the neutrophil culture medium conditioned by cancer cells, was introduced into the lower chamber as a chemotactic agent. After 6 h, the migrated cells in the lower chamber were counted.

For the group co-culturing tumor cells with neutrophils, first, use 500 µl of poly-L-lysine solution (#P4832, Sigma) to evenly coat the bottom of a 24-well plate, and then wash with Ca^2+^/Mg^2+^-free PBS and set aside. Add 3 × 10^5 neutrophils to the lower chamber. For the NETs inhibition group, add DNase I after 15 min. Add 5 × 10^5 cancer cells to the upper chamber, and after 6 h, count the migrating cells in the lower chamber.

### Neutrophil migration analysis

5 × 10^4 neutrophils were added to the upper chamber of a Transwell plate (3462, CORNING), and RPMI 1640 medium containing 10% FBS was added to the lower chamber. Human recombinant MIP-1α protein (11292-H08Y, SinoBiological) or human MIP-1α neutralizing antibody (AF-270-NA, RD) was added to the lower chamber as a chemotactic agent according to each group. After 3 h, the medium in the lower chamber was collected, and the migrated neutrophils in the lower chamber were counted.

### ELISA assay

To detect serum MPO-DNA, the ELISA method mentioned was used with slight modifications. Taking 5 µg ml − 1 anti-MPO monoclonal antibody (ABD Serotec, 0400-0002) as the capture antibody, the 96-well microtiter plate was coated at 4 °C overnight. When the blocking of 1% BSA was finished, we added patient serum and peroxidase-conjugated anti-DNA monoclonal antibody (Component 2 of the Cell Death Detection ELISA Kit, Roche, 11774425001), which were incubated for 2 h at room temperature, and were then washed by PBS three times. We also added peroxidase substrate (Roche, 11774425001). Upon incubation for 40 min at 37 °C, a microplate reader (Infinite M200 PRO) was used to measure optical density at 405 nm.

### Scanning electron microscopy

The slips were covered with neutrophils and were left untreated or treated with 200 nM PMA for 4 h. For the purpose of detecting acellular NETs, isolated NETs were added and coated on the cover slips overnight at 37 °C. Subsequently, as mentioned before, we processed the samples to scan electron microscopy. To be brief, 2.5% glutaraldehyde was used to fix the samples overnight. When fixation was completed, they went through washing via PBS, incubated using 1% osmium tetroxide, dehydrated via the graded series of ethanol, and next experienced critical point drying, after which a 2 nm platinum coating was carried out. Subsequently, we coated the samples by adopting the 5 nm carbon and analyzed them by utilizing the FEI Quanta 200 scanning electron microscope.

### Animal experiments

The female BALB/c mice, which were 6 weeks old and obtained from Liaoning Changsheng (Liaoning, China), were used. All mice were injected with 1 × 10^6 cells in the fourth mammary fat pad, with 4T1 cells being implanted in situ. Treatments targeting c-FOS (T5224, 3 mg/kg) and NETs (DNase I, 300 U/mouse) were administered via intraperitoneal injection daily thereafter. PBS was used as a control. We measured tumor growth every two days by utilizing calipers, with the calculation of tumor volume being 1/2 (length × width²). The 6-week-old female BALB/c mice were used in another mouse model of breast cancer metastasis. We suspended 4T1 (2 × 10^6) cells in 100 µL of HBSS ice solution, followed by their injection through using the tail vein. We also conducted a 2-week monitoring of the mice. The NightOWL II LB983 Imaging System (Berthold) was used to obtain BLI.

### Statistical analysis

The GraphPad Prism 8 (GraphPad Software) was used to carry out each statistical analysis. To compare means between two groups, we conducted unpaired Student’s t-tests and to compare means among three or more groups, and performed one-way ANOVA (Tukey’s test).

## Results

### Increased formation of NETs in breast cancer patients

**Fig. 1:**
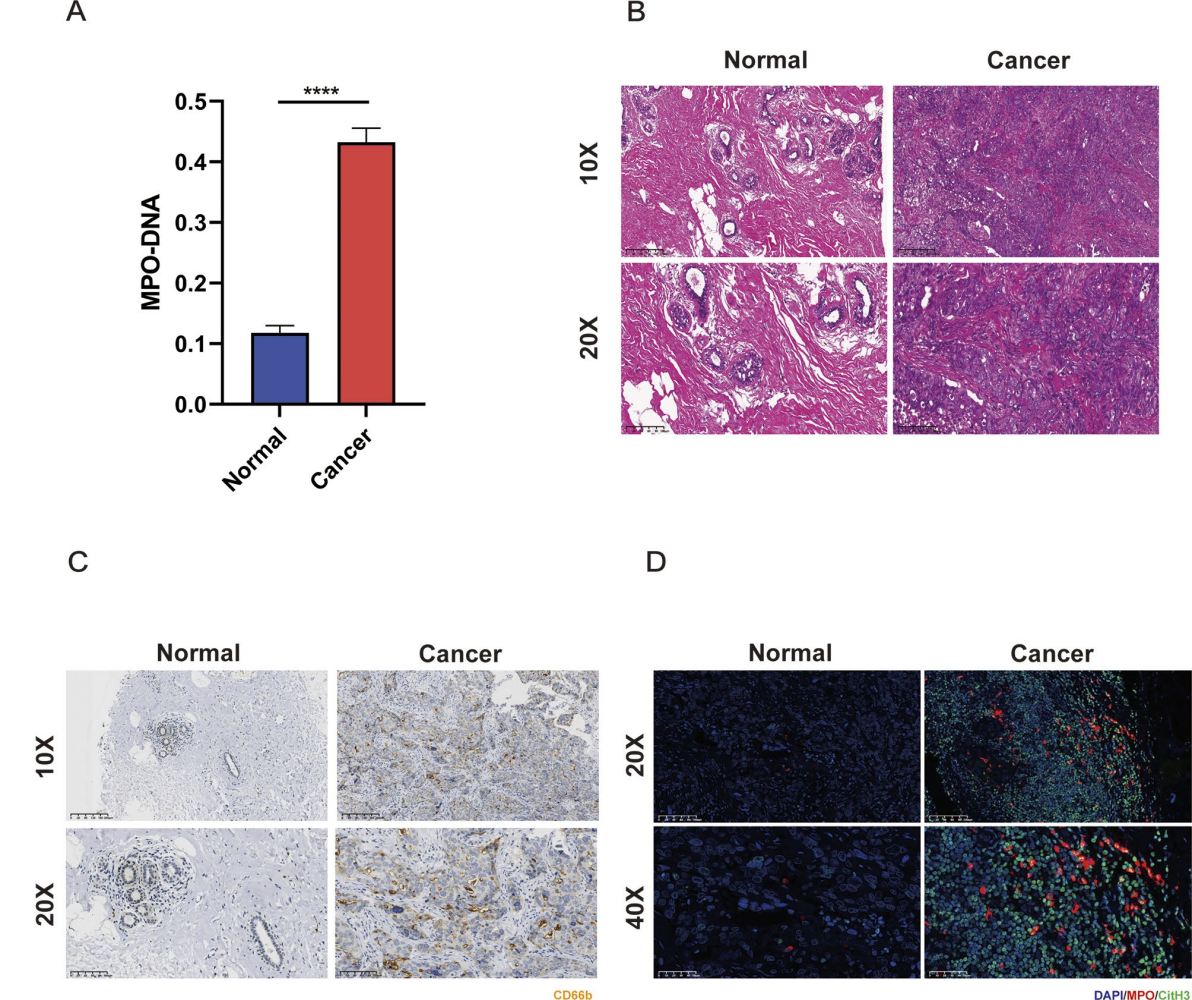
Increased neutrophil infiltration and NETs formation in breast cancer patients. **A** ELISA was used to detect the expression levels of MPO-DNA in the serum of 30 breast cancer patients and healthy individuals. **B** HE staining of the primary lesion and breast tissue in breast cancer. **C** Immunohistochemical staining detects the expression level of CD66B protein in the tissues of breast cancer patients. **D** IF detects the expression levels of MPO (red) and CitH3 (green) proteins in the tissues of breast cancer patients. The data are shown as the means ± SDs; ****P < 0.0001

To explore the correlation between NETs and the occurrence and development of breast cancer, we collected cancerous tissues and matched normal breast tissues from 30 pairs of breast cancer patients, along with paired serum samples. Additionally, we recruited 30 healthy volunteers and collected their serum samples. By utilizing ELISA, MPO: DNA was detected in the patient’s serum, and we also identified that, in comparison with the healthy population, there were greatly increased expression levels of MPO: DNA in the serum of breast cancer patients (Fig. 1A). When expression levels of CD66B in the cancerous and normal breast tissues of breast cancer patients were measured, CD66B expression showed a substantial increase in cancerous tissues (Fig. 1B, C; Tab. S1), which implied increased neutrophil infiltration in the cancerous tissues of breast cancer patients. Furthermore, after immunofluorescent labeling of NETs markers in the tissues, MPO and CitH3 experienced an elevated expression in cancerous tissues in comparison with normal tissues, which suggested that NETs saw an increased formation in the tissues of breast cancer patients (Fig. 1D; Tab. S1).

### c-FOS is an important target gene for NET formation in breast cancer

**Fig. 2:**
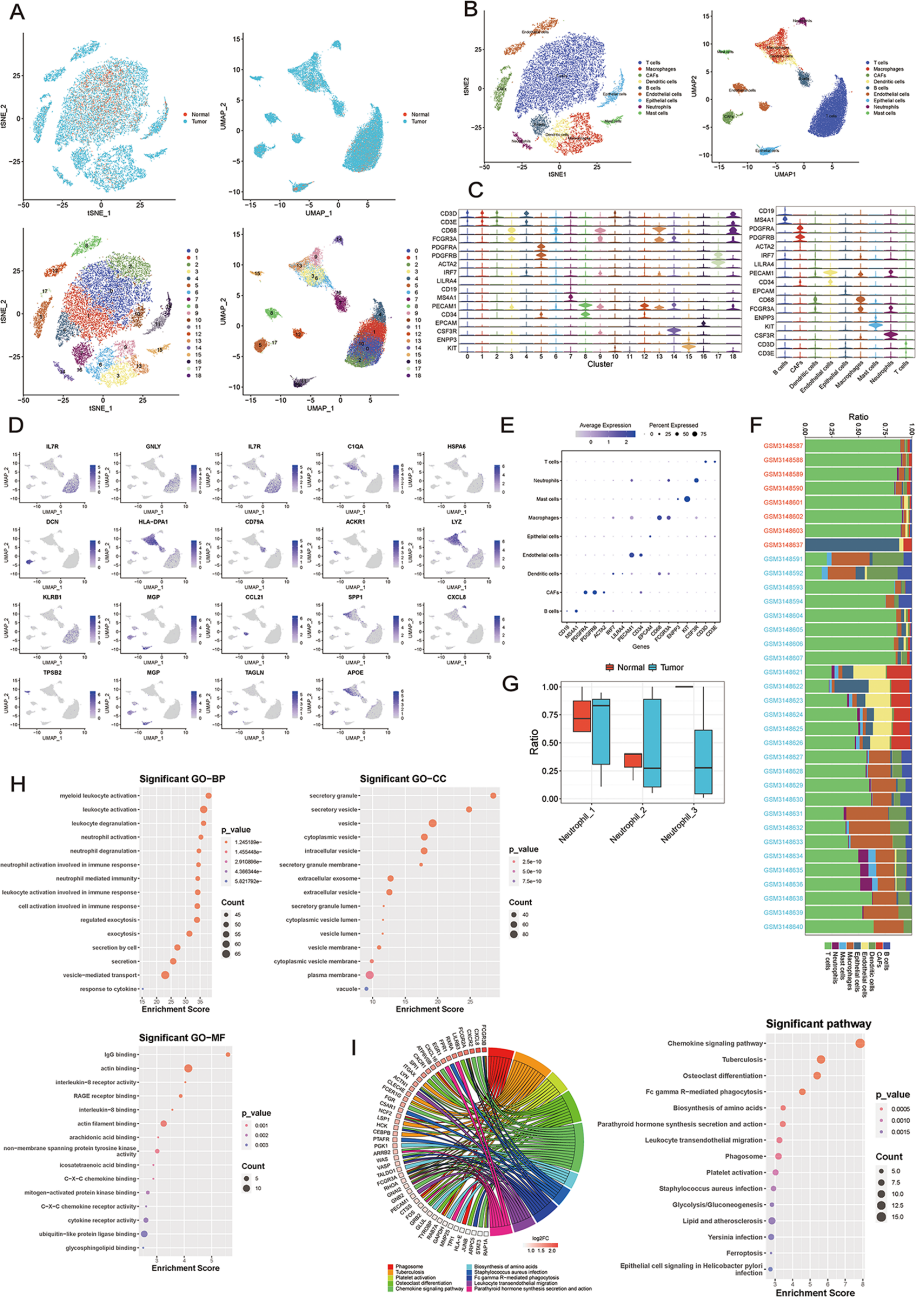
Three clusters of neutrophil genes are associated with breast cancer **A** tSNE and UMAP clustering of selected cells. **B** tSNE and UMAP identification of clustered cell types. **C** Violin plots showing marker gene expression of various cell types. **D** The UMAP plot displays the top marker genes in each cluster. **E** Expression profile of marker genes used for cell identification in various types of cells. **F** Heat maps showing the proportion of various cell types in different samples. **G** Box plots showing the differences in the proportion of cells between different neutrophil clusters. **H** GO enrichment analysis of Neutrophil 1 cluster. **I** KEGG enrichment analysis of Neutrophil 1 cluster

To clarify the specific biological functions of NETs in breast cancer patients, we established a single-cell atlas of breast cancer using single-cell data (GEO: GSE114725). We used UMAP and tSNE methods to cluster and label the tumor cells and normal cells (Fig. 2A). The cell types of each cluster were determined based on the marker genes of the cells (Fig. 2B). We then recalculated the marker genes for each cell type to obtain new marker genes (Fig. 2C-E, Supplementary Fig. S1A). To investigate the differences in cell composition between the breast cancer and normal tissue samples, this study quantified the proportion of each cell type relative to the total cell count in each sample (Fig. 2F). Finally, we conducted further clustering of neutrophils and calculated the specifically expressed genes in each cluster (p < 0.05, FC > 1.5). The results showed that Neutrophil_1 specifically expresses inflammation-related genes such as LITAF, NAMPT, and CXCL8 (Fig. 2G, Supplementary Tab. S2). Using the specifically expressed genes from the three clustered neutrophil clusters, we performed GO and KEGG enrichment analyses. The GO analysis indicated that Neutrophil_1 was significantly enriched in biological functions related to myeloid leukocyte activation, secretory granule, and IgG binding (Fig. 2H). The KEGG analysis revealed significant enrichment of Neutrophil_1 in signaling pathways like chemokine signaling pathway, tuberculosis, osteoclast differentiation (Fig. 2I). Additionally, further analysis of Neutrophil_1 showed specific expression of NETs-related genes such as AQP9, C5AR1, FCGR2A, FCGR3A, FCGR3B, and FPR1 (Supplementary Fig. S1B). It is inferred that Neutrophil_1 is a cluster of pro-tumor neutrophil genes that may be associated with the formation of NETs. Furthermore, GO analysis shows that Neutrophil_2 is significantly enriched in biological functions such as SRP-dependent cotranslational protein targeting to membrane, cytosolic ribosome, and RNA binding (Supplementary Fig. S1C). Neutrophil_3 is significantly enriched in biological functions like regulation of hemopoiesis, cytosol, and mRNA 3’-UTR AU-rich region binding (Supplementary Fig. S1D). KEGG analysis indicates that Neutrophil_2 is significantly enriched in signal pathways including Ribosome, Coronavirus disease-COVID-19, and Antigen processing and presentation (Supplementary Fig. S1E). Neutrophil_3 is significantly enriched in signal pathways associated with Salmonella infection, Coronavirus disease-COVID-19, and Ribosome (Supplementary Fig. S1F). Thus, it is inferred that Neutrophil_2 and Neutrophil_3 are pro-inflammatory neutrophil clusters that may be related to bacterial or viral infections.

**Fig. 3:**
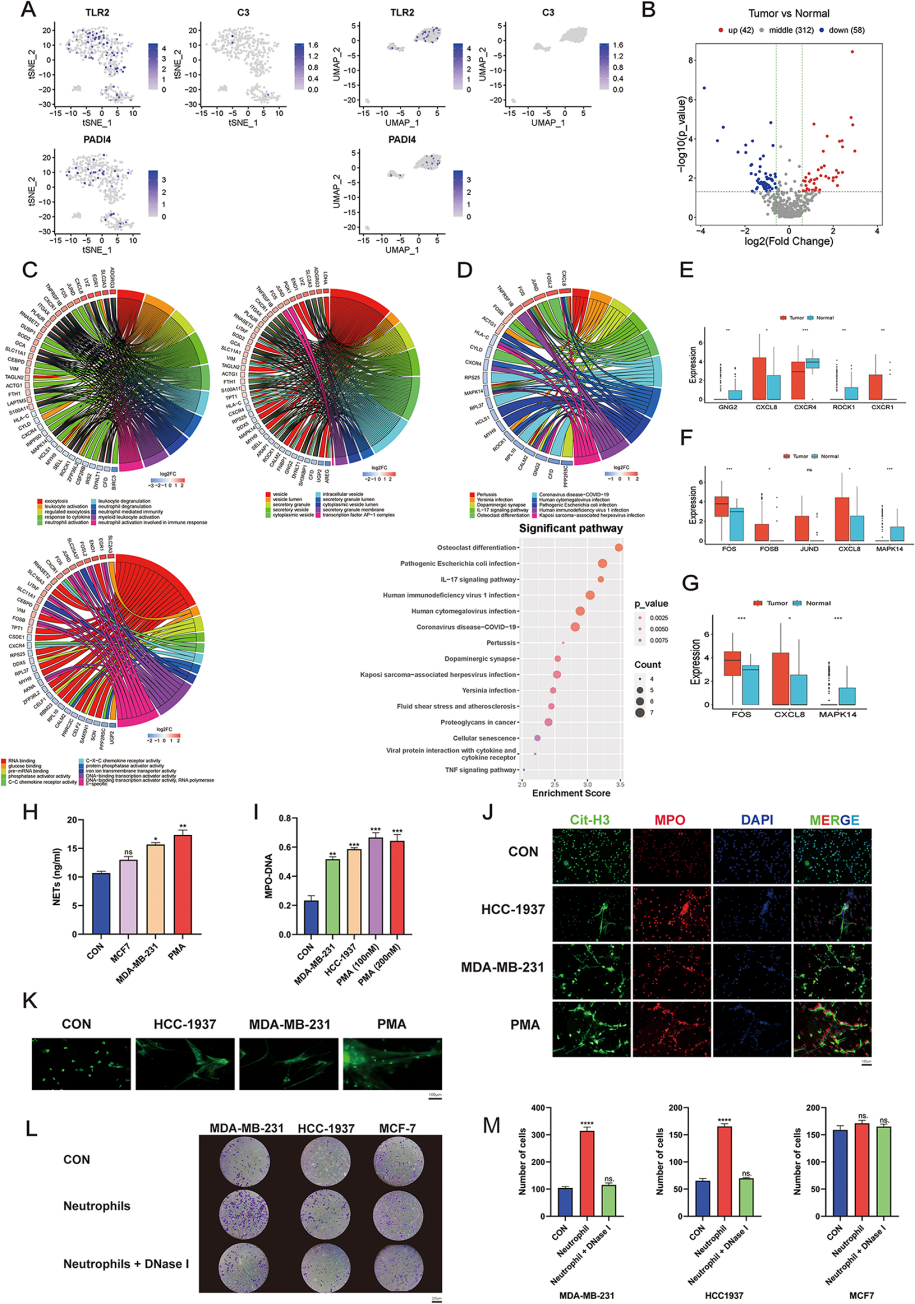
Neutrophil 1 cluster is associated with the formation of NETs in breast cancer. **A** tSNE and UMAP display the expression of NETs-related marker genes. **B** Volcano plot shows differentially expressed genes associated with NETs formation. **C** GO enrichment analysis of differentially expressed genes related to NETs formation. **D** KEGG enrichment analysis of differentially expressed genes related to NETs formation. **E** Differential genes of the Chemokine signaling pathway. **F** Differential genes of the IL-17 signaling pathway. **G** Differential genes of the Toll-like receptor signaling pathway. **H** ELISA detects the expression levels of NETs in the supernatant of neutrophils co-cultured with MCF7 and MDA-MB-231 cells. **I** ELISA detects the MPO-DNA expression levels in the supernatant of neutrophils co-cultured with MDA-MB-231 and HCC-1937 cells. **J** IF detects the expression levels of CitH3 and MPO in neutrophils co-cultured with MDA-MB-231 and HCC-1937 cells. **K** The staining of neutrophils with SYTOX GREEN after co-culture with MDA-MB-231 and HCC-1937 cells. **L**,** M** Assessment of tumor migration ability after co-culture with neutrophils or co-culture with neutrophils in the presence of a NETs inhibitor (DNase I). The data are shown as the means ± SDs; *P < 0.05; **P < 0.01; ***P < 0.001; ****P < 0.0001; ns, nonsignificant

Next, to explore the regulatory mechanisms of NETs in breast cancer, we used NETs marker genes to identify differentially expressed genes (Fig. 3A, B). The results of the GO enrichment analysis for these differential genes indicated a significant association with biological behaviors such as neutrophil activation involved in immune response, transcription factor AP-1 complex, and C-X-C chemokine receptor activity (Fig. 3C). The results of the KEGG enrichment analysis showed that the differential genes were significantly related to pathways such as osteoclast differentiation, pathogenic Escherichia coli infection, IL-17 signaling pathway, and Toll-like receptor signaling pathway (Fig. 3D, Supplementary Tab. S3). Genes such as FOS (c-FOS), CXCL8, and MAPK14 exhibited significant differences in the breast cancer group and were enriched in NETs-related pathways like Chemokine signaling pathway, IL-17 signaling pathway, and Toll-like receptor signaling pathway (Fig. 3E-G, Supplementary Tab. S3). Therefore, we speculate that the intersecting gene c-FOS is an important target gene for the formation of NETs in breast cancer.

### High expression of NETs in TNBC promotes cell migration

The biological functions of NETs in different subtypes of breast cancer were then examined. We co-cultured various breast cancer cells with primary human peripheral blood neutrophils. The results showed that both MCF7 and MDA-MB-231 cells could stimulate an upregulation of NETs expression (Fig. 3H). The triple-negative breast cancer cell lines MDA-MB-231 and HCC1937 were found to induce an upregulation of MPO-DNA, a marker for NETs formation. Additionally, we used PMA to stimulate neutrophils as a positive control for NETs formation. It was found that both 100 nM and 200 nM concentrations of PMA effectively stimulated neutrophils to form NETs (Fig. 3I). Immunofluorescence staining results indicated that the positivity rates of CitH3 and SYTOX GREEN were elevated by co-culturing HCC1937 and MDA-MB-231 cells with neutrophils (Fig. 3J, K). According to the Transwell assay, co-culturing of neutrophils with MDA-MB-231 and HCC-1937 cells significantly promoted breast cancer cell migration compared to co-culturing with MCF7 cells (Fig. 3L). Based on these results, we found that NETs can promote the migration of breast cancer cells and are highly expressed in TNBC.

### c-FOS promotes the formation of NETs in TNBC

**Fig. 4:**
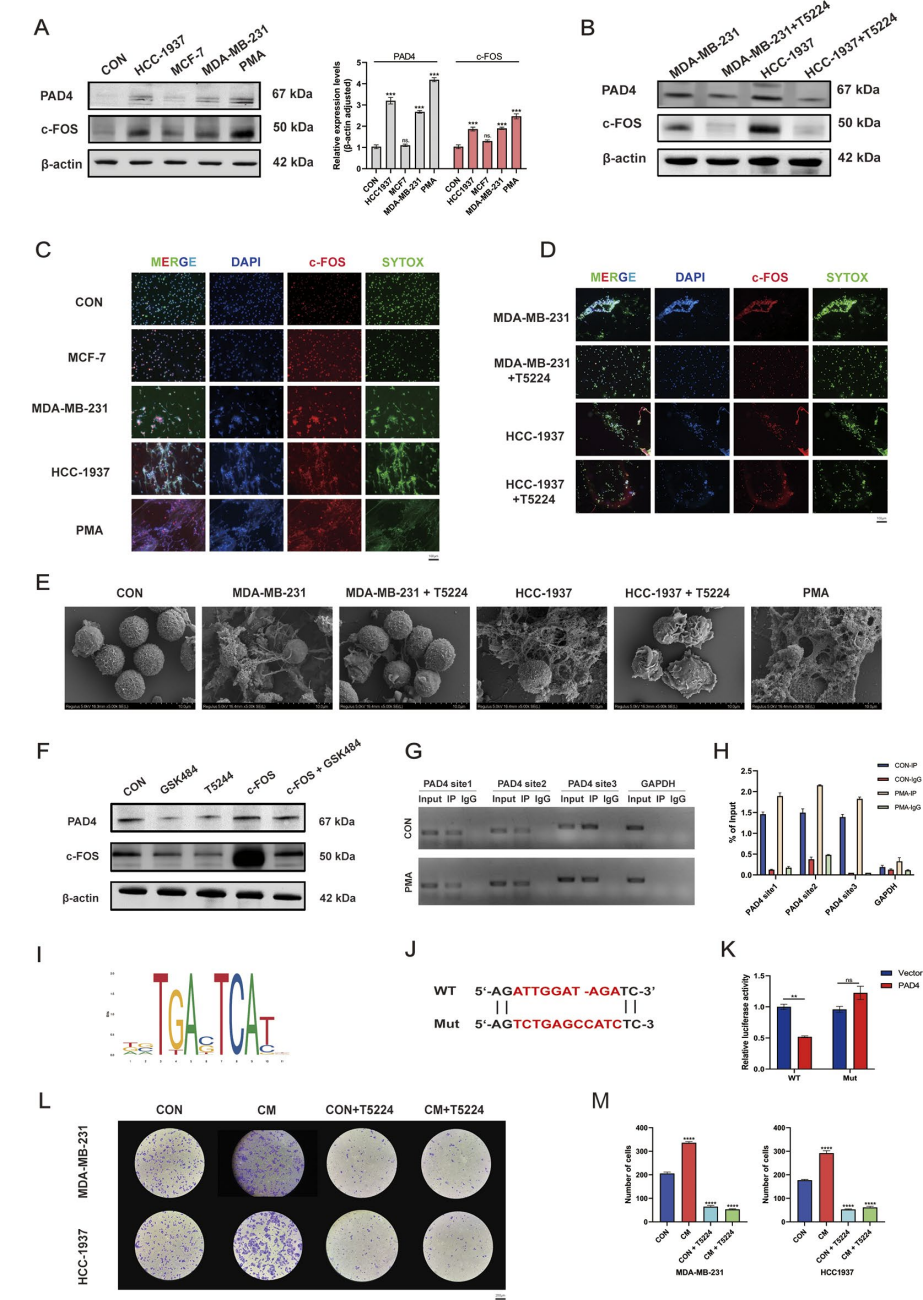
c-FOS is a key gene affecting NETs formation in breast cancer. **A** Detection of protein levels of c-FOS and PAD4 in neutrophils after stimulation with CM from different cell lines. **B** Detection of protein levels of c-FOS and PAD4 in neutrophils after stimulation with CM from different cell lines or CM pre-treated with T5224. **C** IF detection of expression levels of c-FOS and SYTOX GREEN in neutrophils after stimulation with CM from different cell lines. **D** IF detection of expression levels of c-FOS and SYTOX GREEN in neutrophils after stimulation with CM from different cell lines or conditioned media pre-treated with T5224. **E** SEM detection of NETs formation. **F** Detection of protein expression levels of c-FOS and PAD4 in neutrophils after inhibiting PAD4 (GSK484), inhibiting c-FOS (T5224), over-expressing c-FOS (recombinant c-FOS protein), or simultaneous interventions. **G**,** H** ChIP-qPCR detection of the binding ability of c-FOS and PAD4. **I** JASPAR predicts the c-FOS sequence that binds to the PAD4 promoter region. **J** Predicted sequences that bind to the PAD4 promoter region. **K** Detection of the binding region between the c-FOS and PAD4 promoters using a luciferase reporter assay. **L**,** M** T5224 reduced the enhanced tumor migratory ability induced by the neutrophil-conditioned medium (CM). Data are presented as means from three independent experiments ± S.D; **P < 0.01; ***P < 0.001; ****P < 0.0001; ns, nonsignificant

The effect of c-FOS on NET formation in TNBC was explored following the aforementioned single-cell data bioinformatics analysis and molecular biological experiments we investigated. Protein expression levels were measured after stimulating neutrophils through using conditioned media (CM) from a variety of subtypes of breast cancer cells, which revealed high expression of c-FOS in neutrophils stimulated with CM from TNBC subtype cell lines HCC1937 and MDA-MB-231, with a positive correlation with PAD4 expression (Fig. 4A). Next, the MDA-MB-231 and HCC-1937 groups were selected for c-FOS inhibition, and after the addition of the c-FOS inhibitor T5224, the expression of c-FOS in neutrophils significantly decreased, along with a positive correlation with PAD4 (Fig. 4B, Supplementary Fig. S1G, H). According to immunofluorescence detection, the positivity rates of c-FOS and SYTOX GREEN in neutrophils significantly increased after stimulation with MDA-MB-231 and HCC1937 CM (Fig. 4C). After stimulation with MDA-MB-231 and HCC1937 CM pre-treated with T5224, the positive rates of c-FOS and SYTOX GREEN in neutrophils were inhibited (Fig. 4D). In addition, the detection by SEM also found that the formation of NETs increased after stimulation with MDA-MB-231 and HCC1937 CM, which could be suppressed by T5224 (Fig. 4E).Based on these results, it was hypothesized that c-FOS may affect NET formation by directly regulating PAD4. We then performed rescue experiments, which implied that overexpression of recombinant c-FOS protein could effectively restore the reduced PAD4 expression caused by the GSK484 (PAD4 inhibitor) (Fig. 4F). Additionally, the results from the ChIP assay indicated that c-FOS, which acted as a transcription factor, possesses regions that directly bind to the PAD4 promoter (% of input > 1%), and this binding capability can be boosted upon stimulation with PMA (Fig. 4G-H). In addition, we predicted the sequence of the transcription factor c-FOS that binds to PAD4 using the JASPAR database (Fig. 4I). We also confirmed through a luciferase reporter assay that c-FOS can bind to the promoter region of PAD4 (Fig. 4J-K). Finally, tumor migration experiments revealed that inhibiting c-FOS in neutrophils can reduce the promoting effect of neutrophils on the migration of MDA-MB-231 and HCC1937 cells (Fig. 4L-M).

### p38-ROS promotes the formation of NETs in TNBC via c-FOS

**Fig. 5:**
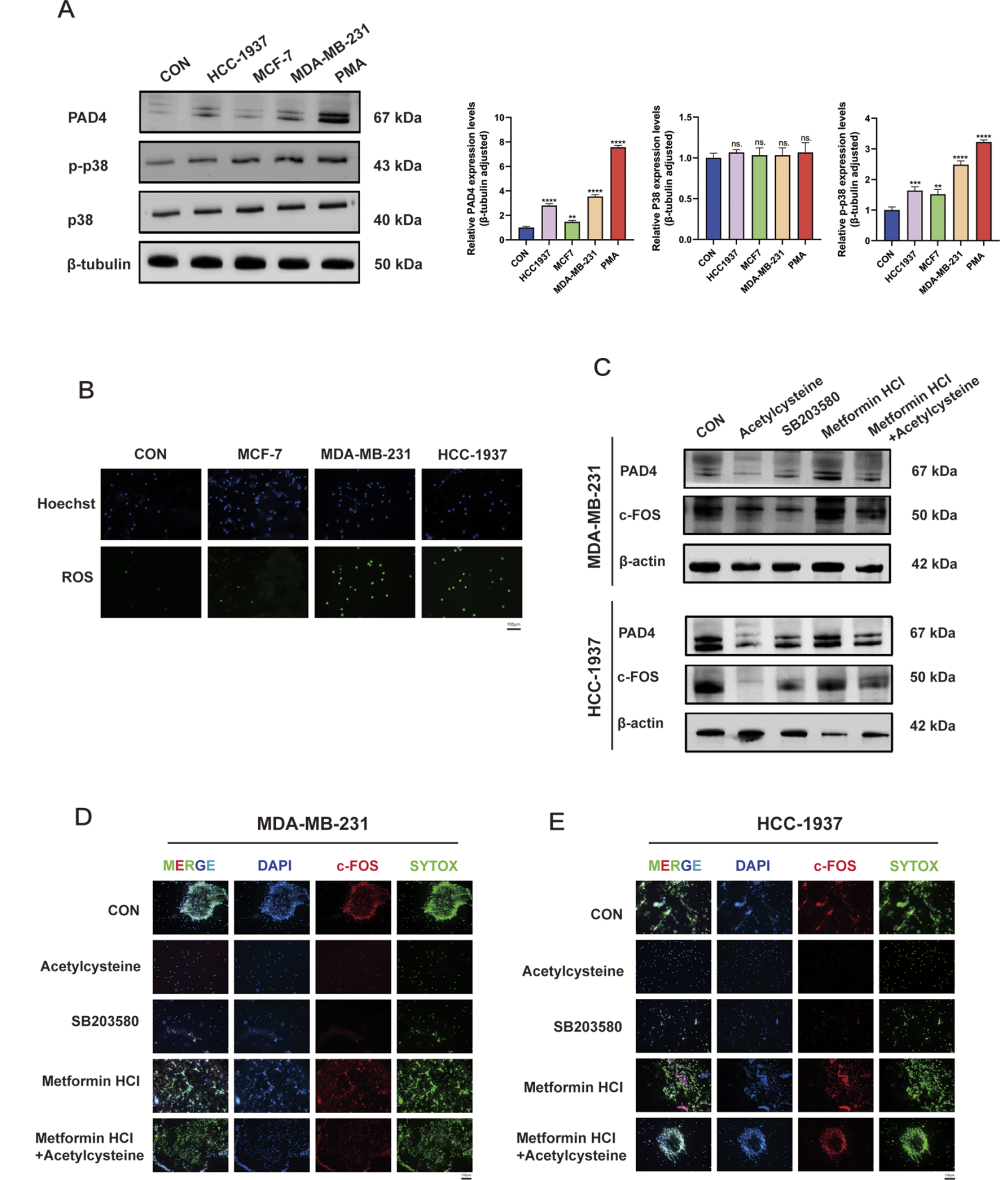
p38-ROS regulates NETs formation through c-FOS. **A** Detection of protein expression levels of PAD4, p38, and p-p38 in neutrophils after stimulation with CM from various cell lines. **B** IF detection of ROS expression levels in neutrophils after stimulation with CM from various cell lines. **C** After simultaneously stimulating neutrophils with MDA-MB-231 or HCC1937 CM and inhibiting ROS (Acetylcysteine), inhibiting the p38 pathway (SB203580), activating the p38 pathway (Metformin HCl), or through combined interventions, detection of protein expression levels of PAD4 and c-FOS in neutrophils. **D**,** E** After simultaneously stimulating neutrophils with MDA-MB-231 or HCC1937 CM and inhibiting ROS (Acetylcysteine), inhibiting the p38 pathway (SB203580), activating the p38 pathway (Metformin HCl), or through combined interventions, IF detection of expression levels of c-FOS and SYTOX GREEN in neutrophils. Data are presented as means from three independent experiments ± S.D. **P < 0.01; ***P < 0.001; ****P < 0.0001; ns, nonsignificant

Also, the KEGG and GO enrichment results for NET-related genes in Neutrophil 1 indicated that c-FOS expression is associated with the p38 (MAPK14)-ROS signaling pathway (Fig. 3E-G). Therefore, we examined the expression of proteins in neutrophils stimulated by different breast cancer cell-conditioned media (CM). The results showed that after 2 h of stimulation with TNBC CM, the expression of p-p38 in neutrophils was upregulated and positively correlated with PAD4 expression (Fig. 5A). Immunofluorescence detection of ROS indicated that after 2 h of stimulation with breast cancer cell CM, the expression of ROS in neutrophils was upregulated (Fig. 5B). Furthermore, compared to MCF7 CM, ROS levels in neutrophils were significantly increased after stimulation with MDA-MB-231 and HCC1937 CM (Fig. 5B). Therefore, we selected MDA-MB-231 and HCC1937 cells for rescue experiments. The results showed that using the ROS inhibitor (Acetylcysteine) or the p38 inhibitor (SB203580) alone effectively reduced the expression levels of c-FOS and PAD4 proteins. Additionally, when p38 agonist (Metformin HCl) was used to activate the p38-MAPK pathway while inhibiting ROS, this situation could be reversed (Fig. 5C). Cell immunofluorescence detection revealed that inhibiting ROS or p38 alone could significantly reduce the positivity rates of c-FOS and SYTOX GREEN, while activating the p38-MAPK pathway during ROS inhibition can reverse the aforementioned effects (Fig. 5D, E).

### c-FOS promotes neutrophil recruitment via factors such as MIP-1α

**Fig. 6:**
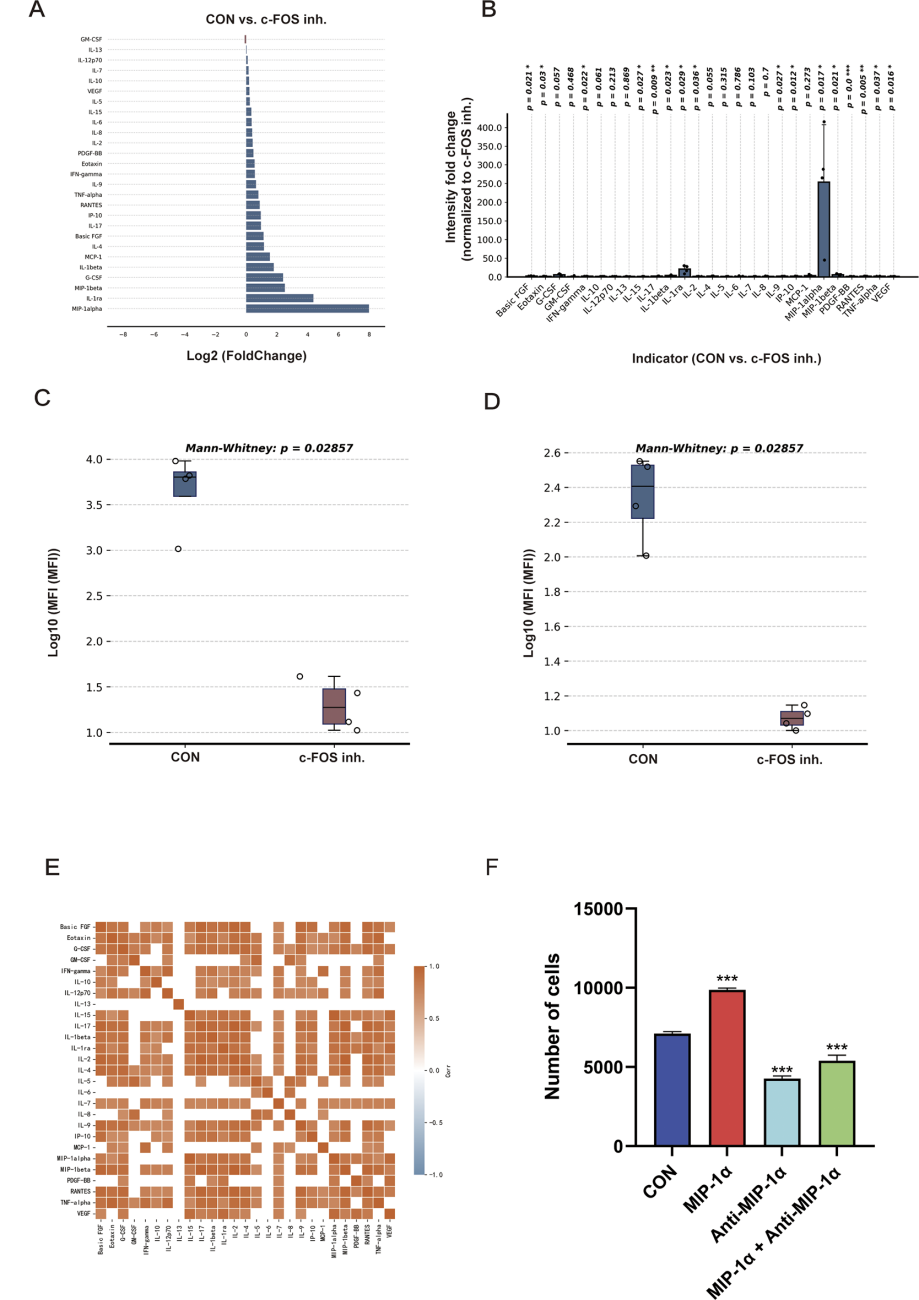
Inhibition of c-FOS can reduce the expression of inflammatory factors in neutrophils. **A**,** B** Luminex detects the expression levels of cytokines after c-FOS inhibition. **C** Luminex detects the expression levels of MIP-1α after c-FOS inhibition. **D** Luminex detects the expression levels of IL-1ra after c-FOS inhibition. **E** Heat map showing the correlation of inflammatory cytokines between the two groups. **F** Assessment of the effect of MIP-1α on neutrophil migratory ability

Next, how c-FOS influences neutrophil recruitment was investigated. Interestingly, the KEGG and GO enrichment results for Neutrophil 1 also indicated that c-FOS affects the expression of CXCL8 and CXCR4 and some other inflammatory factors (Figs. 2H-I and 3E-G). This suggests that c-FOS may affect neutrophil recruitment by exerting an impact on the secretion of certain chemokines. Through Luminex multiplex cytokine detection, after inhibiting the expression of c-FOS in neutrophils, we found that the expression of various inflammatory factors, including MIP-1α, IL-1ra, MIP-1β, and G-CSF, was significantly downregulated (Fig. 6A). Among these, the downregulation of MIP-1α and IL-1ra was most pronounced (Fig. 6B). Furthermore, we conducted a univariate analysis of different inflammatory factors, and the results showed that there were significant statistical differences in the expression of MIP-1α and IL-1ra (Fig. 6C, D; Supplementary Figure S2). At the same time, the study found a strong correlation between the expression of MIP-1α and IL-1ra (Fig. 6E). Furthermore, we selected MIP-1α, which exhibited the most significant change in expression levels, for intervention experiments. Neutrophil migration assays revealed that using recombinant MIP-1α protein as a chemoattractant could promote neutrophil migration, and the combined use of MIP-1α neutralizing antibodies could reverse this effect (Fig. 6F). These data indicate that c-FOS in tumor-associated neutrophils can regulate MIP-1α and IL-1ra secretion and therefore recruit more neutrophils to metastatic sites and form a pre-metastatic niche.

### Targeting c-FOS with T5224 treatment effectively controls the progression of TNBC and neutrophil infiltration

**Fig. 7:**
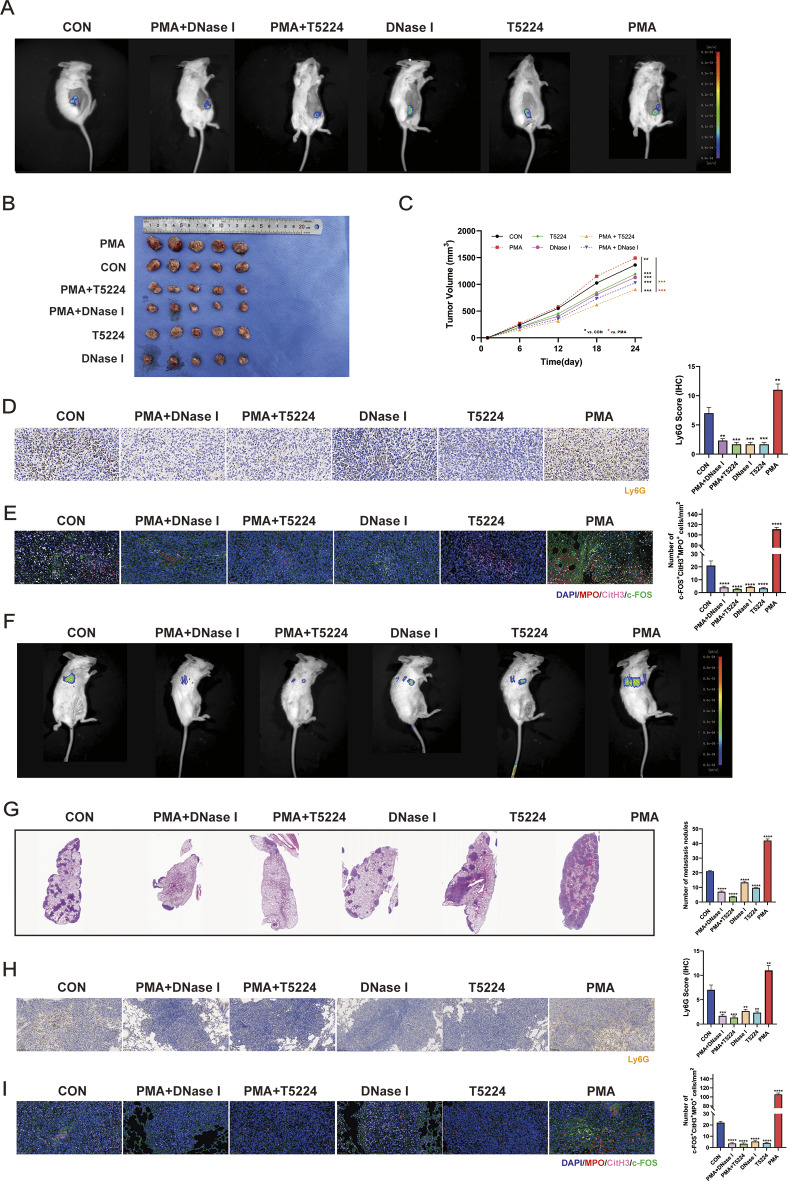
Targeting c-FOS treatment can inhibit tumor growth and lung metastasis. **A** The luminescence intensity of tumor cells grown in mice treated with targeted c-FOS (T5224) or NETs (DNase I). **B**,** C** Tumor volumes were determined on the indicated days with different treatments in 4T1 mice (*n* = 5). **D** Immunohistochemical staining detects the expression levels of Ly6G protein in mouse tumor tissues. **E** IF detects the expression levels of MPO (red), CitH3 (pink), and c-FOS (green) proteins in mouse tumor tissues. **F** Luminescence intensity of lung metastatic tumor cells in mice treated with targeted c-FOS or NETs. **G** Lung metastasis nodules of the 4T1 tumor were measured after treatment. **H** Immunohistochemical staining detects the expression levels of Ly6G protein in mouse lung metastatic tumor tissues. **I** IF detects the expression levels of MPO (red), CitH3 (pink), and c-FOS (green) proteins in mouse lung metastatic tumor tissues. Data are presented as means from three independent experiments ± S.D. **P < 0.01; ***P < 0.001; ****P < 0.0001

**Fig. 8:**
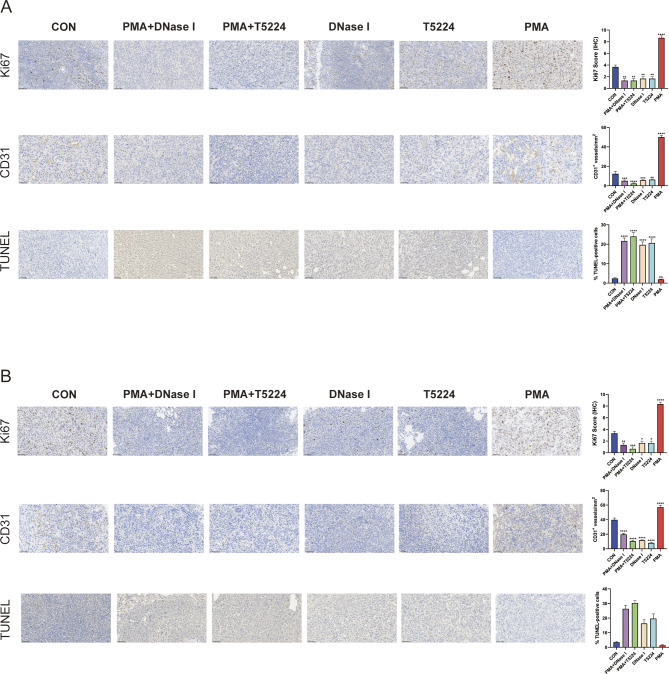
Targeting c-FOS affects tumor proliferation, vascular density, and tumor apoptosis. **A** Representative images and analysis results of Ki67, CD31, and TUNEL staining in mouse tumor tissue. **B** Representative images and analysis results of Ki67, CD31, and TUNEL staining in lung metastatic tumor tissue of mice

**Fig. 9:**
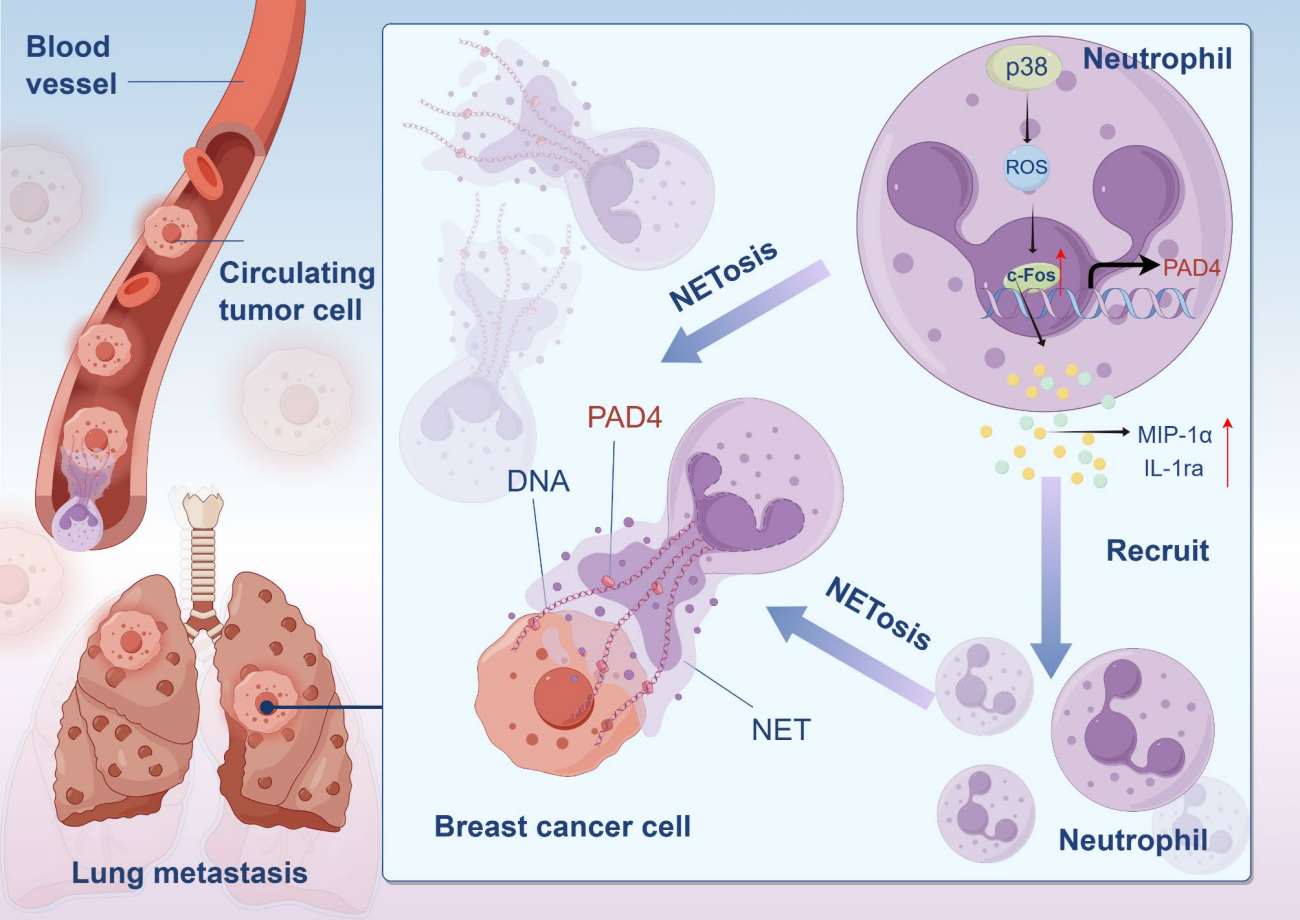
Mechanistic of c-FOS promoting neutrophil recruitment and NETs formation in triple-negative breast cancer

Since c-FOS contributes a lot to breast cancer progression and its clinical relevance, we aimed to assess whether targeting c-FOS might be efficient for treating breast cancer progression. First of all, 4T1 cells were utilized to construct an in situ tumor model of triple-negative breast cancer, and intraperitoneal injection was leveraged to administer the c-FOS inhibitor T5224 or the NETs inhibitor DNase I. The results indicated that both T5224 and DNase I treatments effectively slowed tumor growth (Fig.s 7 A-C). We identified that T5224 exhibited better therapeutic efficacy in models pre-treated with PMA. Immunohistochemical staining revealed a significant decrease in the positivity rate of Ly6G in PMA-pretreated mice (Fig. 7D). This trend was also observed in the immunofluorescence staining results for tissue MPO, CitH3, and c-FOS (Fig. 7E). Next, the tail vein injection of 4T1 cells was used to build a lung metastasis model of triple-negative breast cancer, followed by its treatment through employing T5224. The study found that both T5224 alone and T5224 treatment after PMA pre-treatment effectively alleviated breast cancer lung metastasis (Fig. 7F, G). In comparison with the control group, there was a significant decrease in the positivity rates of Ly6G, MPO, CitH3, and c-FOS in the metastatic tumors of the treatment group (Fig. 7H, I). Finally, to clarify the mechanism of c-FOS targeted therapy in tumor growth and lung metastasis, we assessed tumor proliferation ability, vascular density, and cell apoptosis. The results indicated that c-FOS targeted therapy effectively reduced the Ki67 positive rate in tumors, inhibited angiogenesis, and enhanced tumor cell apoptosis (Fig. 8A). Additionally, a similar trend was observed in lung metastatic tumors (Fig. 8B). To sum up, the data verify that c-FOS contributes to the regulation of tumor-associated neutrophils, which implies that c-FOS is likely to function as the therapeutic target for lung metastasis in breast cancer (Fig. 9).

## Discussion

NET is crucial for the mediation of the pro-tumor activity of neutrophils, with DNA, serving as their main component, and therefore can directly drive cancer cell proliferation [4, 24]. In the prior research, it was found that CCDC25 is a NET-DNA sensor on the surface of breast cancer cells, and that colon cancer cell proliferation can be driven by the interaction between NETs triggered by SKAP1 and CCDC25 [4, 25]. Recent research has found that NETs have immunosuppressive activity within cancer, and NET suppression promotes the efficacy of immune checkpoint blockade therapy [26]. NETs can protect tumor cells from immune-mediated cytotoxicity since they can establish a physical barrier that protects tumor cells from contacting immune effector cells [27]. Moreover, the physical barrier mediated by NETs helps exclude T cells from tumors, and the density of NETs has a negative relationship with the CD8^+^ T cell density in the tumor microenvironment [28, 29]. Apart from being a physical barrier, NET is likely to induce T cell exhaustion and dysfunction as well and therefore impair their anti-tumor function [30, 31]. NETs also exert inhibitory effects on the cytotoxicity of NK cells, which is another crucial effector cell type that is included within the anti-tumor immune responses. Studies have shown that the therapeutic effect of adoptive NK cell transfer can be greatly strengthened by inhibiting NETs in colon cancer cells [25]. In this research, based on single-cell bioinformatics analysis, in vivo experiments, and in vitro assays, it is demonstrated that c-FOS can promote the formation of NETs in TNBC. Additionally, the downregulation of c-FOS effectively inhibits the NET formation and hence leads to controlled tumor growth and lung metastasis. Therefore, c-FOS may alter the pulmonary tumor microenvironment by mediating NET formation. The recent findings show that c-FOS-mediated transcriptional activation can lead to both radiation resistance in TNBC and the inactivation of CD8^+^ T cells [32]. According to Wang et al., the downregulation of c-FOS within CAR-T cells can suppress genes related to T cell effector functions, glycolysis, and apoptosis [33]. Furthermore, c-FOS can induce the expression of PD-1 through transcriptional activation. This research also confirmed that being a classical transcription factor, c-FOS can directly bind to regions in the PAD4 promoter and activate transcription. Since PAD4 is a structural component of NETs, it is proven that c-FOS can directly regulate the formation of NETs in breast cancer-associated neutrophils. To sum up, we speculate that c-FOS may promote lung metastasis in TNBC by directly regulating the NET formation and therefore can realize the restructuring of the tumor immune microenvironment.

Mechanistically, we found that inhibiting the p38 MAPK (p38) - ROS pathway can downregulate c-FOS and therefore further inhibit NET formation. In the prior research, it was found that through the interaction with CXCR2 on neutrophils, IL-8 derived from diffuse large B-cell lymphoma (DLBCL) can form NETs via the Src, p38, and ERK signaling pathways [28]. Furthermore, it has been found that by utilizing the DAMP molecule HMGB1 on the neutrophils surface, TRAPs can activate the TLR4-Myd88-p38/ERK axis and then induce NET formation [34]. In this study, a novel NET formation mechanism was offered, in which the p38-ROS-cFOS-PAD4 axis can directly induce NET formation, and blocking this axis inhibits NET formation.

Additionally, powerful immunostimulatory properties are shown in NETs. Owing to the NETs-TLRs interaction, the expression of a variety of target genes is initiated, such as inflammatory factors, immunosuppressive factors and chemokines [35–39]. The single-cell sequencing bioinformatics analysis in this study found that CXCL8 (IL-8) expression was associated with c-FOS, which was consistent with previous studies that indicated that IL-8 could induce NETs, elevating the cascade effect exerted by NETs on cancer [40–43]. Moreover, we confirmed through Luminex assays that c-FOS also primarily stimulates the expression of MIP-1α and IL-1ra. Through those inflammatory mediators, the immune microenvironment can be further reformed to jointly support the progression of cancer.

Also called CCL3, macrophage inflammatory protein 1α (MIP-1α) refers to a ligand for CCR1, CCR3, and CCR5, and is also an important pro-inflammatory factor that contributes much to the immune system, especially functioning as the chemokine for a variety of lymphocytes [44–47]. A significant secretion of CCL3, CCL2, CCL4, CXCL1, and CXCL8 exists in acute myeloid leukemia (AML) cells [48]. There is higher nuclear factor-κB (NF-κB) activity in those AML cells, explaining the reason why those chemokines see an increase in production [49]. This may also explain the changes in various inflammatory factors observed after inhibiting c-FOS in this study. Additionally, research has found that the JAK1-STAT1 pathway can stimulate macrophages to secrete CCL3, which in turn damages the tight junction of the gastric epithelium through phosphorylation of P38 [50]. Thus, it is speculated that the increased secretion of CCL3 caused by elevated c-FOS expression may further activate the p38-cFOS-PAD4 axis, which can promote a malignant cycle of NET formation.

Being the IL-1 superfamily member, interleukin-1 receptor antagonist (IL-1ra) is expressed in the liver, leukocytes and placenta. Compared to normal cells, IL-1ra not only witnessed a substantial elevation on the surface of leukemia and cancer cells but also acted as a promising cancer immunotherapy target [51–53]. In recent research, it has been identified that IL-1ra is a therapeutic target for AML and solid tumors [54, 55]. IL-1ra contributes centrally to signaling and amplifying IL-1 family cytokines, such as IL-1, IL-33 and IL-36) and to amplifying and transmitting some other membrane proteins, such as C-kit and cystine transporters and tyrosine kinase receptors including Fms-related receptor tyrosine kinase 3 (FLT3) [51–58]. Immunotherapies targeting IL-1ra are being developed for solid tumors [59, 60]. To treat AML, monoclonal IL-1ra antibodies (nadunolimab) have been tested in clinical trials by combining with some other immunotherapies targeting CAR-T cells or as a single agent [61]. Recent studies have found that therapies targeting IL-1ra can activate T cells and guide their involvement in lysing AML cells [62]. Reports indicate that IL-1ra has a relationship with rapid disease progression and poor prognosis, and also seen the high expression on the surface of AML, high-risk myelodysplastic syndrome (MDS) cells and chronic myeloid leukemia (CML) [63, 64]. High IL-1ra expression may promote malignant cell growth by amplifying inflammatory and oxidative stress-driven mechanisms, which is correlated with significantly poorer clinical outcomes [57]. In this study, it is implied that high expression of c-FOS may upregulate IL-1ra. This may lead researchers to speculate that this regulation can further recruit neutrophils with the potential to form NETs, amplifying inflammatory and oxidative stress-driven mechanisms, thereby promoting NET formation and TNBC growth and establishing a pre-metastatic niche within the lungs.

However, some limitations of this study should be acknowledged. First, while targeting c-FOS therapy can reduce neutrophil infiltration and NETs formation in breast cancer, we did not investigate changes and interactions of other immune cells, such as T cells, within the immune environment, which requires further research in the future. Second, we utilized a multiparametric detection method to identify various inflammatory factors that c-FOS may regulate, but due to the complex regulation of inflammatory factor secretion by cells, we cannot determine the role of any single inflammatory factor in the c-FOS/NETs formation pathway. Finally, although the mouse model and 4T1 cells can simulate the development and lung metastasis of human breast cancer, the limitations posed by interspecies differences between tumor cells and neutrophils should be considered. Further research is needed, along with validation of these findings in clinical studies.

## Conclusion

In summary, this study indicates that c-FOS can increase the formation of NETs in TNBC through the ROS-p38-cFOS-PAD4 axis. This pathway also promotes the expression of MIP-1α and IL-1ra, as well as other inflammatory factors, facilitating the recruitment of neutrophils to create a pre-metastatic niche. Targeting c-FOS therapy can improve the tumor microenvironment, thereby inhibiting tumor growth and lung metastasis, but further research and clinical trials are still needed. Therefore, targeting the c-FOS/NETs pathway provides a theoretical reference for discovering immunotherapeutic targets in TNBC.

## Electronic supplementary material

Below is the link to the electronic supplementary material.

Supplementary Material 1: Supplementary Fig. 1 Neutrophil cluster is associated with the formation of NETs in breast cancer. **A** The heatmap displays the top 10 new marker genes for various cell types. **B** The violin plot shows the NETs-related genes in Neutrophil 1. **C** GO enrichment analysis of differentially expressed genes associated with Neutrophil 2. **D** GO enrichment analysis of differentially expressed genes associated with Neutrophil 3. **E** KEGG enrichment analysis of differentially expressed genes associated with Neutrophil 2. **F** KEGG enrichment analysis of differentially expressed genes associated with Neutrophil 3. **G** Expression levels of PAD4 mRNA in neutrophils stimulated with MDA-MB-231 CM and the c-FOS inhibitor (T5224). **H** Expression levels of PAD4 mRNA in neutrophils stimulated with HCC-1937 CM and the c-FOS inhibitor (T5224).

Supplementary Material 2: Supplementary Fig. 2 Inhibition of c-FOS can reduce the expression of inflammatory factors in neutrophils. A Luminex detection of expression levels of Basic FGF, Eotaxin, G-CSF, GM-CSF, IFN-gamma, IL-1beta, IL-2, IL-4, IL-5, IL-6, IL-7, IL-8, IL-9, IL-10, IL-12p70, IL-13, IL-15, IL-17, IP-10, MCP-1, MIP-1beta, PDGF-BB, RANTES, TNF-alpha, and VEGF after c-FOS inhibition

Supplementary Material 3

## Data Availability

No datasets were generated or analysed during the current study.

## Abbreviations

NETs: Neutrophil extracellular traps
TNBC: Triple-negative breast cancer
CTSC: Cysteine protease C
AP-1: Activator protein 1
CSCs: Cancer stem cells
bZIP: Basic region leucine zipper
PMA: Phorbol 12-myristate 13-acetate
UMIs: Unique molecular identifiers
CM: Conditioned media
IHC: Immunohistochemical Staining
TSA: Tyramide signal amplification
DLBCL: Diffuse large B-cell lymphoma
MIP-1α: Macrophage inflammatory protein 1α
AML: Acute myeloid leukemia
IL-1ra: Interleukin-1 receptor antagonist
FLT3: Fms-related receptor tyrosine kinase 3
MDS: Myelodysplastic syndrome
CML: Chronic myeloid leukemia
