# Supplementary figures and images for: C-FOS promotes the formation of neutrophil extracellular traps and the recruitment of neutrophils in lung metastasis of triple-negative breast cancer
Source: J Exp Clin Cancer Res. 2025 Mar 28;44:108. doi: 10.1186/s13046-025-03370-2 (PMC11951605; doi:10.1186/s13046-025-03370-2)

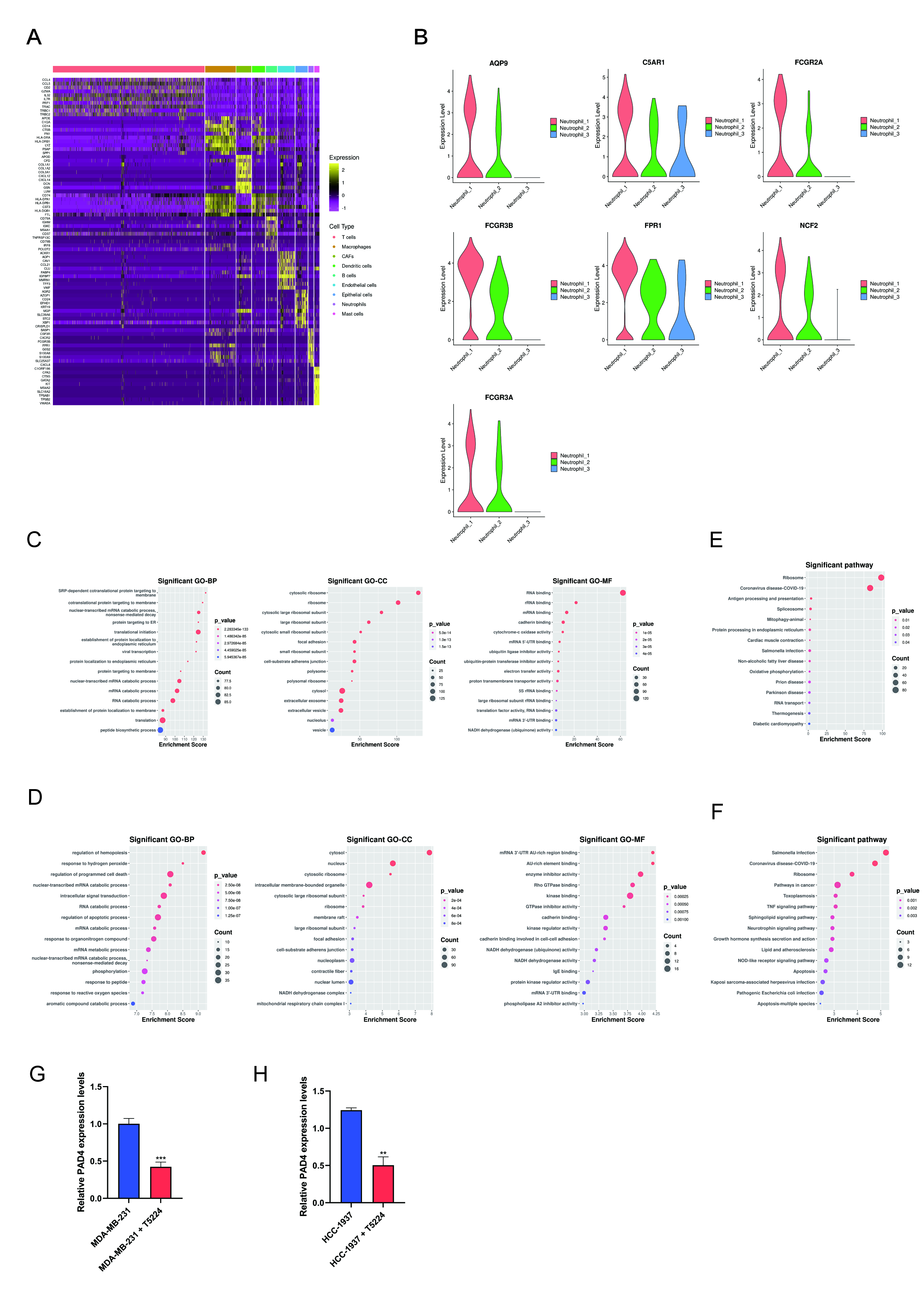

Supplement: Supplementary file 1 — Supplementary Material 1: Supplementary Fig. 1 Neutrophil cluster is associated with the formation of NETs in breast cancer. A The heatmap displays the top 10 new marker genes for various cell types. B The violin plot shows the NETs-related genes in Neutrophil 1. C GO enrichment analysis of differentially expressed genes associated with Neutrophil 2. D GO enrichment analysis of differentially expressed genes associated with Neutrophil 3. E KEGG enrichment analysis of differentially expressed genes associated with Neutrophil 2. F KEGG enrichment analysis of differentially expressed genes associated with Neutrophil 3. G Expression levels of PAD4 mRNA in neutrophils stimulated with MDA-MB-231 CM and the c-FOS inhibitor (T5224). H Expression levels of PAD4 mRNA in neutrophils stimulated with HCC-1937 CM and the c-FOS inhibitor (T5224). [file 13046_2025_3370_MOESM1_ESM.tif]

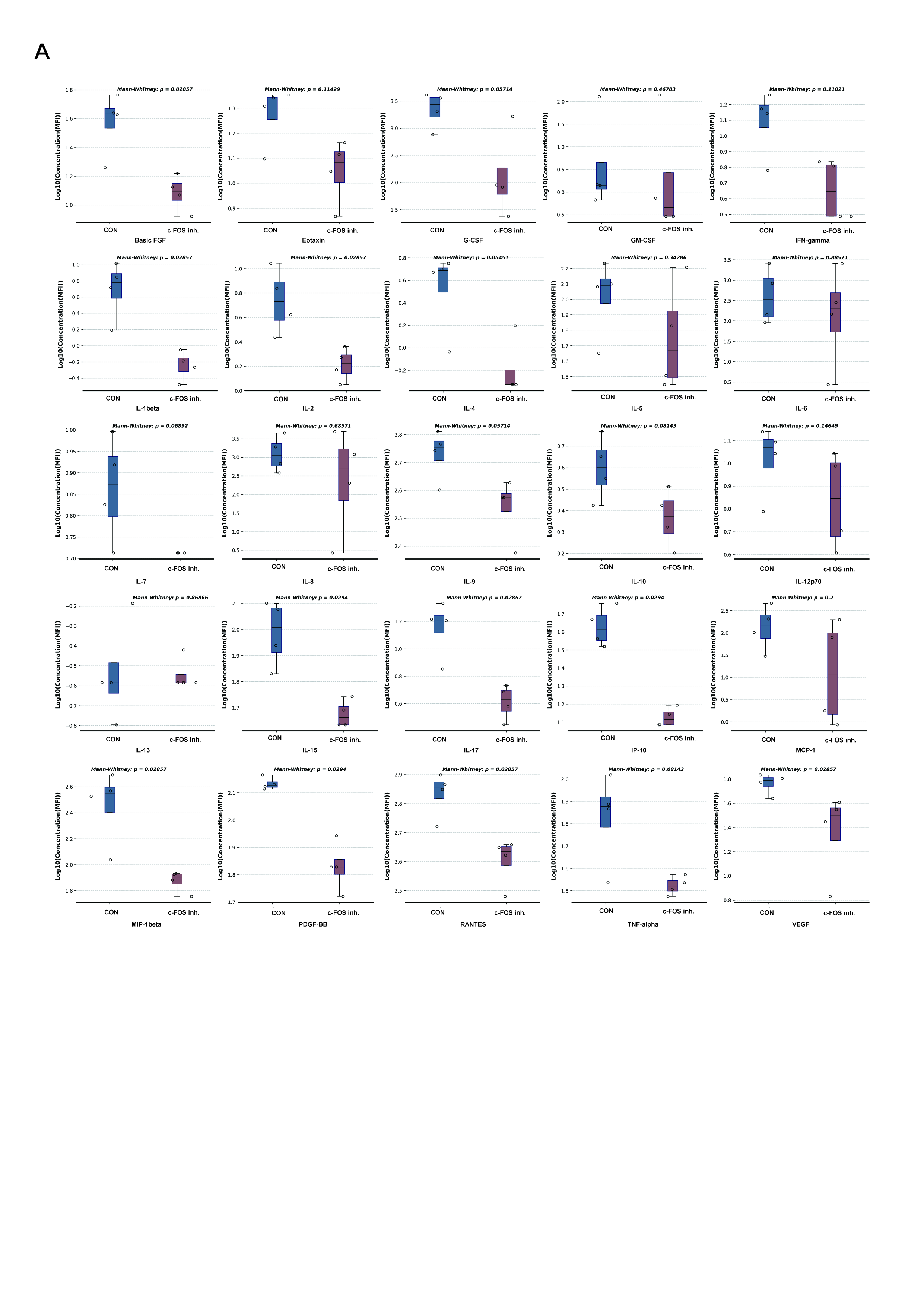

Supplement: Supplementary file 2 — Supplementary Material 2: Supplementary Fig. 2 Inhibition of c-FOS can reduce the expression of inflammatory factors in neutrophils. A Luminex detection of expression levels of Basic FGF, Eotaxin, G-CSF, GM-CSF, IFN-gamma, IL-1beta, IL-2, IL-4, IL-5, IL-6, IL-7, IL-8, IL-9, IL-10, IL-12p70, IL-13, IL-15, IL-17, IP-10, MCP-1, MIP-1beta, PDGF-BB, RANTES, TNF-alpha, and VEGF after c-FOS inhibition [file 13046_2025_3370_MOESM2_ESM.tif]
