# Supplementary material for: C-FOS promotes the formation of neutrophil extracellular traps and the recruitment of neutrophils in lung metastasis of triple-negative breast cancer
Source: J Exp Clin Cancer Res. 2025 Mar 28;44:108. doi: 10.1186/s13046-025-03370-2 (PMC11951605; doi:10.1186/s13046-025-03370-2)
Supplement: Supplementary file 3 — Supplementary Material 3 [file 13046_2025_3370_MOESM3_ESM.docx]

**Table1** Characteristics of CD66B and NETs in 30 pairs of patients

|  | CD66B score (IHC) | | NETs score (IF)^*^ | |
| --- | --- | --- | --- | --- |
| Patients | Cancer | Normal | Cancer | Normal |
| 1 | 4 | 0 | 105 | 1 |
| 2 | 6 | 2 | 110 | 0 |
| 3 | 4 | 0 | 130 | 2 |
| 4 | 6 | 1 | 100 | 1 |
| 5 | 4 | 0 | 123 | 2 |
| 6 | 4 | 1 | 134 | 3 |
| 7 | 4 | 1 | 156 | 1 |
| 8 | 4 | 1 | 122 | 0 |
| 9 | 6 | 1 | 100 | 0 |
| 10 | 4 | 1 | 166 | 1 |
| 11 | 4 | 0 | 99 | 0 |
| 12 | 4 | 1 | 125 | 1 |
| 13 | 6 | 1 | 134 | 3 |
| 14 | 6 | 1 | 128 | 2 |
| 15 | 9 | 2 | 134 | 4 |
| 16 | 4 | 0 | 123 | 0 |
| 17 | 8 | 1 | 144 | 3 |
| 18 | 4 | 1 | 147 | 1 |
| 19 | 4 | 1 | 130 | 3 |
| 20 | 6 | 0 | 140 | 3 |
| 21 | 8 | 1 | 130 | 4 |
| 22 | 6 | 0 | 133 | 2 |
| 23 | 4 | 1 | 136 | 2 |
| 24 | 4 | 1 | 145 | 3 |
| 25 | 4 | 1 | 126 | 1 |
| 26 | 4 | 1 | 138 | 3 |
| 27 | 6 | 1 | 127 | 0 |
| 28 | 4 | 1 | 142 | 4 |
| 29 | 4 | 0 | 152 | 2 |
| 30 | 9 | 1 | 122 | 2 |

*NETs score: number of MPO^+^CitH3^+^cells/mm^2^

**Table2** Gene names in 3 neutrophil clusters

| Neutrophil-1 | Neutrophil-2 | Neutrophil-3 |
| --- | --- | --- |
| **LITAF,** **NAMPT,** **CXCL8,** **FOS,** FCGR3B, IFITM2, SLC25A37, NEAT1, S100A11, FPR1, CSF3R, G0S2, CEBPB, S100A9, DUSP1, SOD2, MCL1, FTH1, SAT1, MT-RNR2, RNF149, ALOX5AP, GAPDH, CXCR2, FMNL1, IVNS1ABP, MXD1, LAPTM5, TAGLN2, SRGN, BCL2A1, FTL, LSP1, UBALD2, RGS2, FLOT1, GNAI2, SLC11A1, PNRC1, LYN, ZFP36L1, CEBPD, H3F3B, TNFRSF1B, FCGR2A, SLC16A3, VASP, RHOA, BASP1, KIAA1551, PGK1, LRRFIP1, CAP1, NINJ1, TYROBP, ENO1, MYO1F, RHOG,  SLA, ITM2B, ATP6V0B, TPI1, SORL1, ZYX, ANXA11, FAM49B, SDCBP, C5AR1, FAM65B, OAZ1, EFHD2, C10ORF54, ADGRG3, HCLS1, LCP1, LRP10, ITGAX, MT-RNR1, MIDN, ARHGAP26, RNASET2, CLEC4E, IER3, XPO6, CD37, SPI1, AQP9, ICAM3, COTL1, TREM1, LILRB3, PHC2, WSB1, LDHA, MBOAT7, UBE2R2, SLC2A3, ARPC5, YPEL3, SERPINA1, GNB2, GLUL, HLA-E, CTSS, UBE2B, RXRA, SMAP2, SERPINB1, CXCR1, TALDO1, RAP1A, NCF2, SELL, HCK, WAS, BCL6, FAM129A,  ARRB2, JUNB, GABARAP, RP11-463O12.5, AMICA1, VNN2, RAB7A, EVI2B, BRI3, STAT3, PELI1, ST1, YPEL5, CFLAR, FFAR2, PRR13, R3HDM4, GCA, IER2, PLAUR, FCER1G, APOBEC3A, RP4-635A23. 6, MX2, ACTN1, IL1R2, TSC22D3, CMTM6, GPSM3, PECAM1, GMFG, LINC01272, C4ORF3, EGR1, CD82, CXCL16, MSRB1, STK4, ABTB1, TRIB1, KPNB1, AIF1, GRB2, ACSL1, CPPED1, VMP1, RTN3, MNDA, RBPJ, PTAFR, RTN4, ANP32A, SEC14L1, FCGR3A, FGR, IL17RA, S100A12, ARHGAP9, SYAP1, ANPEP, MMP25 | MT-CO1, RPS16, RPS18, EEF1A1, RPS25, RPL13, RPLP2, RPL10, RPS8, RPS4X, RPS29, RPS12, RPL27A, RPL3, RPL18, RPS24, RPS15A, RPL37A, RPS23, RPL7A, RPL12, RPS20, RPL28, RPS19, RPS27, RPS2, RPL23A, RPS28, RPS14, RPL30, MT-CO2, RPL38, RPL19, RPL15, MT-ATP6, RPL4, RPL13A, MT-CYB, RPL37, MT-CO3, RPL14, RPL8, RPL32, RPL23, RPSA, RPL35, RPL18A, RPLP1, RPS3A, TRAC, HNRNPA1, RPS21, RPL31, RPL24, RPL11, AHNAK, RPL26, GNB2L1, HSPA8, RPL35A, RPS27A, RPS3, RP11-425L10.1, RPL5, RPL10A, IL32, RPS10, CCL5, RPS15, RPS6, RPS17, MT-ND4, TRBC2, RPS7, RPL9, CTC-575D19.1, RPLP0, RPS13, RPL7, COX7C, EEF1B2, RPL27, HSP90AA1, RPL34, RP11-832N8.1, TMSB10, RPL6, RPL13AP5, RPL36, XIST, AC016708.2, RPL29, HSP90AB1, RPS5, CTD-3035D6.1, CD52, FYN, 6-Sep, EVL, PTMA, MT-ND5, PPIA, EEF2, NACA, ETS1, CD2, MT-D2, GLTSCR2, RPS11, MT-ND3, RPS23P8, RPL41, ITK, FXYD5, AC004453.8, RPL3P4, IL7R, CD74, RPL21, RGS1, RPL39, S100A10, RPS9, RPS26, CLEC2D, DUSP2, RP5-1056L3.3, RPL22, ANXA1, RP11-234A1.1, BTF3, TOMM7, NAP1L1, APOBEC3G, CD99, DDX5, PPDPF, CD3D, SPOCK2, MT2A, CALR, RPL18AP3, DNAJB1, MTATP6P1, PTGER4, FAU, YBX1, UBB, CD48, HINT1, HCST, FAM102A, CD69, EIF4B, KLRD1, ZFP36L2, ID2, UBA52, GZMA, GNLY, PABPC1, UCP2, FLNA, RPSAP58, NPM1, PRDM1, RPS11P5, EIF3E, TMC8, CD81, MYL12A, UQCRB, RPL17, RPS7P10, RPL13P12, CITED2, MT-ND1, SNHG6, RPL36AL, PCSK7, LDHB, AES, COX4I1, RP11-51O6.1, RP11-112J1.1, EIF4A2, HNRNPA2B1, RP11-475C16.1, SNHG5, RGCC, ARL4C, MT-ATP8, IFI44L, TMSB4XP4, CCL4, HMGB1, HNRNPDL, IKZF1, CHCHD2, HNRNPF, PHIP, RUNX3, PPP1CA, VIM, CDC37, PIK3IP1, SRSF7, GSTP1, NDUFA4, AC016739.2, SRSF2, MGEA5, ERP29, SET, 9-Sep, SNRPD2, ATP5L, AIM1, TAGAP, ANXA2, JUN, ATXN7, RPS28P7, P4HB, TMEM123, HNRNPL, CAST, N4BP2L2, RBM3, BTN3A2, MBNL1, COX7A2, EIF3H, UBE2I, HERPUD1, HMGN1, PNISR, ISCU, UBE2Q2P6, TRIM56, ACTG1, SUN2, HSPA1A, COX6A1, ARF6, LYZ, ATP5G2, HLA-DRA, COX6B1, SRSF3, TRIM22, TRA2B, IFITM1, RNF213, SH3BGRL, SETD5, FOXP1, MTND1P23, EIF5, ISG20, SF3B1, ZNF207, | AREG, CLC, FCER1A, GATA2, BIRC3, MS4A3, HDC, ZFP36L2, MT-ND2, RUNX1, SOCS1, EREG, SAMSN1, UGP2, FAM101B, ALAS1, MS4A2, SCCPDH, CTNNBL1, PLD3, CXCR4, AKAP12, CFD, RHOH, EMP3, MTATP6P1, PAG1, KLF2, CD69, MT-ATP8, XBP1, DCTN6, MAP3K1, GPX1, CSF2RB, CLSTN1, DDX5, TNFAIP3, JAK2, MT-ATP6, VPS53, SLC35E1, DUSP6, BCL2L11, ANXA1, PRNP, HK3, CTSB, PDLIM5, PRKX, RPLP1, CASP3, LAIR1, GABPB1-AS1, MYH9, ZBTB20, ARL5A, FBXL20, ANKRD28, PCSK7, BTG1, RPLP2, MT-ND1, CCNI, RAB31, LIMD2, PTMA, USF2, ANXA2, KCNE1, CDC42SE2, IRS2, TPST2, RPS6KA3, FNBP1, HSH2D, PCM1, HIST1H4C, METAZOA-SRP, MAF, CAMK1D, CAPG, HIST1H1C, TMEM164, ROCK1, ARHGEF6, PIM1, FLNA, SELK, C1ORF162, RABAC1, MT-ND4, UBB, TACC1, CEP350, HLX, PNRC2, G3BP2, LINC01578, CELF2, RHBDD2, HNRNPD, GNAQ, DEK, KPNA4, USP15, PTP4A2, SRSF11, RPS11, PPP2R1A, ABI1, ZFP36, TXNIP, OIP5-AS1, GPR65, MT-ND5, CALM1, CEP63, NFKBIA, MORF4L1, CYBA, RPS29, OSM, CLK1, LAPTM4A, NFKBIZ, ETS1, MAPK14, EIF1, IQGAP2, HNRNPA0, RNF19B, RPSA, RPSAP58, PSEN1, IRF1, TCEB2, RPL10, NCKAP1L, N4BP2L2, HNRNPH1, TLE4, ADD3, RAB27A, RBM3, KMT2E, AHNAK, ATXN7, NFE2L2, CD63, MALAT1, AES, PPP1R15A, SON, RPL39, RPL15, STK17B, CCNDBP1, HNRNPDL, CDC42SE1 |

**Table3** KEGG analysis of neutrophil 1

| ID | Term | Count | *P* value | FDR |
| --- | --- | --- | --- | --- |
| hsa04380 | Osteoclast_differentiation | 6 | 0.000333106 | 0.039791556 |
| hsa05130 | Pathogenic_Escherichia_coli_infection | 7 | 0.00059177 | 0.039791556 |
| hsa04657 | IL-17_signaling_pathway | 5 | 0.000628288 | 0.039791556 |
| hsa05170 | Human_immunodeficiency_virus_1_infection | 7 | 0.000914236 | 0.043426194 |
| hsa05163 | Human_cytomegalovirus_infection | 7 | 0.001294197 | 0.048919172 |
| hsa05171 | Coronavirus_disease-COVID-19 | 7 | 0.001544816 | 0.048919172 |
| hsa05133 | Pertussis | 4 | 0.002358128 | 0.061671214 |
| hsa04728 | Dopaminergic_synapse | 5 | 0.002857209 | 0.061671214 |
| hsa05167 | Kaposi_sarcoma-associated_herpesvirus_infection | 6 | 0.002931713 | 0.061671214 |
| hsa05135 | Yersinia_infection | 5 | 0.003354859 | 0.061671214 |
| hsa05418 | Fluid_shear_stress_and_atherosclerosis | 5 | 0.003570439 | 0.061671214 |
| hsa05205 | Proteoglycans_in_cancer | 6 | 0.003949634 | 0.062535878 |
| hsa04218 | Cellular_senescence | 5 | 0.00582397 | 0.085119559 |
| hsa04061 | Viral_protein_interaction_with_cytokine_and_cytokine_receptor | 4 | 0.006317619 | 0.085739115 |
| hsa04668 | TNF_signaling_pathway | 4 | 0.009370408 | 0.105555391 |
| hsa05131 | Shigellosis | 6 | 0.00945167 | 0.105555391 |
| hsa04670 | Leukocyte_transendothelial_migration | 4 | 0.00995806 | 0.105555391 |
| hsa05132 | Salmonella_infection | 6 | 0.009999984 | 0.105555391 |
| hsa04621 | NOD-like_receptor_signaling_pathway | 5 | 0.010739041 | 0.107390407 |
| hsa00010 | Glycolysis/Gluconeogenesis | 3 | 0.013284276 | 0.118309679 |
| hsa04062 | Chemokine_signaling_pathway | 5 | 0.013605512 | 0.118309679 |
| hsa05031 | Amphetamine_addiction | 3 | 0.014378557 | 0.118309679 |
| hsa04622 | RIG-I-like_receptor_signaling_pathway | 3 | 0.014944381 | 0.118309679 |
| hsa05120 | Epithelial_cell_signaling_in_Helicobacter_pylori_infection | 3 | 0.014944381 | 0.118309679 |
| hsa04210 | Apoptosis | 4 | 0.018045354 | 0.137144687 |
| hsa04010 | MAPK_signaling_pathway | 6 | 0.021176273 | 0.149073719 |
| hsa05417 | Lipid_and_atherosclerosis | 5 | 0.02118416 | 0.149073719 |
| hsa04810 | Regulation_of_actin_cytoskeleton | 5 | 0.022341397 | 0.15160234 |
| hsa04261 | Adrenergic_signaling_in_cardiomyocytes | 4 | 0.02485079 | 0.15798044 |
| hsa04610 | Complement_and_coagulation_cascades | 3 | 0.02494428 | 0.15798044 |
| hsa04921 | Oxytocin_signaling_pathway | 4 | 0.027045128 | 0.163942576 |
| hsa04932 | Non-alcoholic_fatty_liver_disease | 4 | 0.027611381 | 0.163942576 |
| hsa04912 | GnRH_signaling_pathway | 3 | 0.031443419 | 0.181037868 |
| hsa04713 | Circadian_entrainment | 3 | 0.034995536 | 0.184216895 |
| hsa04530 | Tight_junction | 4 | 0.036290345 | 0.184216895 |
| hsa05219 | Bladder_cancer | 2 | 0.036880908 | 0.184216895 |
| hsa04933 | AGE-RAGE_signaling_pathway_in_diabetic_complications | 3 | 0.037790813 | 0.184216895 |
| hsa04144 | Endocytosis | 5 | 0.038398912 | 0.184216895 |
| hsa05142 | Chagas_disease | 3 | 0.039716354 | 0.184216895 |
| hsa05200 | Pathways_in_cancer | 8 | 0.041157849 | 0.184216895 |
| hsa04064 | NF-kappa_B_signaling_pathway | 3 | 0.041691192 | 0.184216895 |
| hsa04620 | Toll-like_receptor_signaling_pathway | 3 | 0.041691192 | 0.184216895 |
| hsa04625 | C-type_lectin_receptor_signaling_pathway | 3 | 0.041691192 | 0.184216895 |
| hsa04928 | Parathyroid_hormone_synthesis_secretion_and_action | 3 | 0.043715026 | 0.18876943 |
| hsa04066 | HIF-1_signaling_pathway | 3 | 0.046841924 | 0.197777013 |
